# An In Vivo EGF Receptor Localization Screen in *C. elegans* Identifies the Ezrin Homolog ERM-1 as a Temporal Regulator of Signaling

**DOI:** 10.1371/journal.pgen.1004341

**Published:** 2014-05-01

**Authors:** Andrea Haag, Peter Gutierrez, Alessandra Bühler, Michael Walser, Qiutan Yang, Maeva Langouët, David Kradolfer, Erika Fröhli, Christina J. Herrmann, Alex Hajnal, Juan M. Escobar-Restrepo

**Affiliations:** 1 University of Zurich, Institute of Molecular Life Sciences, Zurich, Switzerland; 2 PhD program in Molecular Life Sciences, Uni ETH Zürich, Switzerland; University of California San Diego, United States of America

## Abstract

The subcellular localization of the epidermal growth factor receptor (EGFR) in polarized epithelial cells profoundly affects the activity of the intracellular signaling pathways activated after EGF ligand binding. Therefore, changes in EGFR localization and signaling are implicated in various human diseases, including different types of cancer. We have performed the first *in vivo* EGFR localization screen in an animal model by observing the expression of the EGFR ortholog LET-23 in the vulval epithelium of live *C. elegans* larvae. After systematically testing all genes known to produce an aberrant vulval phenotype, we have identified 81 genes regulating various aspects of EGFR localization and expression. In particular, we have found that ERM-1, the sole *C. elegans* Ezrin/Radixin/Moesin homolog, regulates EGFR localization and signaling in the vulval cells. ERM-1 interacts with the EGFR at the basolateral plasma membrane in a complex distinct from the previously identified LIN-2/LIN-7/LIN-10 receptor localization complex. We propose that ERM-1 binds to and sequesters basolateral LET-23 EGFR in an actin-rich inactive membrane compartment to restrict receptor mobility and signaling. In this manner, ERM-1 prevents the immediate activation of the entire pool of LET-23 EGFR and permits the generation of a long-lasting inductive signal. The regulation of receptor localization thus serves to fine-tune the temporal activation of intracellular signaling pathways.

## Author Summary

Abnormal signaling by the epidermal growth factor receptor (EGFR) contributes to the development of various human diseases, including different cancer types. One important mechanism that controls intracellular signal transduction is by regulation of the subcellular receptor localization in the signal-receiving cell. We are investigating the regulation of the EGFR homolog LET-23 in the Nematode *C. elegans* by observing the localization of the EGFR in the epithelial cells of live animals. This approach has allowed us to study the dynamics of receptor trafficking in cells embedded in their natural environment and receiving physiological concentrations of various extracellular signals. In a systematic RNA interference screen, we have identified 81 genes controlling EGFR localization and signaling in different subcellular compartments. One new regulator of EGFR signaling identified in this screen encodes the Ezrin Homolog ERM-1. We show genetic and biochemical evidence indicating that ERM-1 is part of a buffering mechanism to maintain a pool of immobile EGFR in the basolateral membrane compartment of the epithelial cells. This mechanism permits the generation of a long-lasting EGFR signal during multiple rounds of cell divisions. The control of receptor localization is thus necessary for the precise temporal regulation of signal transduction during animal development.

## Introduction

The formation of epithelial tissues involves the polarized distribution of growth factor receptors that determine cell proliferation and differentiation. Notably, changes in EGFR localization have a major impact on signaling and organogenesis [1]–[3].

**Figure 1:**
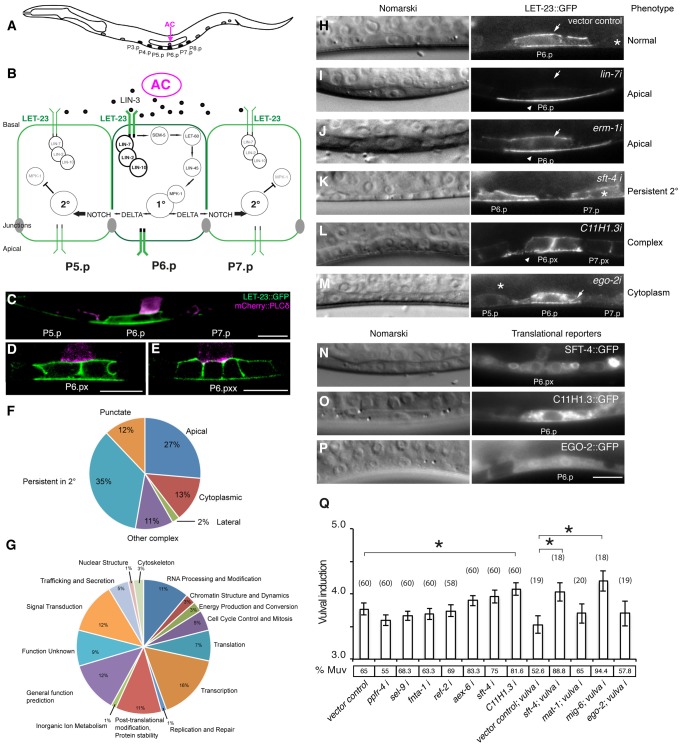
Identification of genes regulating LET-23 EGFR localization and signaling. (A) Schematic drawing of an L2 larva with the location of the VPCs and AC. P5.p, P6.p and P7.p get induced to form the mature vulva. P3.p, P4.p and P8.p divide once and fuse to the hypodermis. (B) Overview of the LET-23 EGFR and NOTCH signaling network controlling 1° and 2° vulval fate specification. (C) LET-23::GFP expression (green) in P6.p of a late L2 larva during vulval induction. The AC is labeled with an *mCherry*::*plcδ^PH^* reporter (magenta) [35]. Note the low LET-23::GFP levels in the 2° P5.p and P7.p. (D) Expression of the LET-23::GFP reporter in the 1° lineage at the Pn.px and (E) Pn.pxx stage. (F) Pie charts indicating the frequencies of the different classes of mislocalization phenotypes observed after RNAi and (G) the Clusters of Orthologous Groups (KOGs) of the 81 genes identified in the screen (H–M). Examples of different genes identified in the LET-23 localization screen. Left panels show the corresponding Nomarski images and right panels LET-23::GFP expression in the 1° cells and their neighbors (asterisks). (H) The negative empty vector control and (I) *lin-7* RNAi as positive control. (J) *erm-1* RNAi as an example for reduced basolateral (arrow) and increased apical localization (arrow head), (K) *sft-4* RNAi with normal localization in P6.p but persistent expression in P7.p (asterisk), and (L) *C11H1.3* RNAi (Pn.px stage) with punctate apical accumulation (arrow head). (M) *ego-2* RNAi with cytoplasmic accumulation of LET-23::GFP in P6.p (arrow head) and P5.p (asterisk). (N) perinuclear localization of SFT-4::GFP in the vulval cells and the AC and (O) intracellular punctate expression of C11H1.3::GFP in P6.p. (P) Cytoplasmic and nuclear expression of EGO-2::GFP in P6.p. (Q) Vulval induction in *let-60(n1046gf)* larvae treated with different RNAi clones. Vulval induction (VI) indicates the average number of induced VPCs per animal. “vulva i” indicates Pn.p cell-specific RNAi in the *rde-1(lf);let-60(n1046gf); [P_lin-31_::rde-1]* background. %Muv (Multivulva) indicates the fraction of animals with VI>3. The numbers of animals scored are indicated in brackets. * Indicates p<0.05 as determined in a two tailed student's t-test - two-sample unequal variance. *t-test* values in *RNAi: C11H1.3* (0.003), *sft-4* (0.013), *mig-6* (0.002). Error bars represent the standard error of the mean. The scale bars are 10 µm.

In *C. elegans*, the *let-23* gene encodes the sole member of the EGFR/ErbB family of receptor tyrosine kinases. *let-23* is involved in a variety of developmental processes including the induction of the hermaphrodite vulva [4]. In early second stage (L2) larvae, LET-23 is expressed at equal levels in the six equivalent vulval precursor cells (VPCs) (P3.p through P8.p) (**Figure 1A**) [5], [6]. Beginning in the L2 stage, the gonadal anchor cell (AC) secretes the EGF ortholog LIN-3, which binds to LET-23 on the basolateral plasma membrane of the VPCs to activate the LET-60 RAS/MPK-1 MAPK signaling pathway [4] (**Figure 1B**). In order to reach high levels of receptor activity, LET-23 must be retained on the basolateral membrane of the VPCs by a ternary protein complex consisting of the PDZ-domain proteins LIN-2 CASK, LIN-10 MINT and LIN-7 VELIS. LIN-7 directly binds to the C-terminal PDZ binding motif of LET-23 [5]. The VPC that is nearest to the AC, P6.p, receives most of the inductive LIN-3 signal and hence adopts the primary (1°) cell fate. P6.p then produces several DELTA ligands, which induce via the NOTCH pathway the secondary (2°) cell fate in the neighboring VPCs P5.p and P7.p [7], [8] (**Figure 1B**). NOTCH signaling blocks RAS/MAPK signaling and results in the endocytosis and degradation of LET-23 in the 2° VPCs [9]–[11]. The distal VPCs P3.p, P4.p and P8.p, which receive only little inductive signal, down-regulate LET-23 expression and adopt the tertiary (3°), uninduced cell fate. As the pathway components are conserved, the study of vulval induction of the worm can be used to find new core components or those required for fine-tune the signaling output. For this purpose, we performed the first systematic *in vivo* screen for regulators of LET-23 EGFR localization and expression in live *C. elegans* larvae.

Through this approach, we have identified 81 genes causing a variety of LET-23::GFP mislocalization phenotypes upon RNAi knock-down. A subset of these genes also controls the strength of the LET-23 EGFR signal produced in the VPCs. We have identified ERM-1, the homologue of mammalian Ezrin, Radixin and Moesin proteins, as a temporal regulator of LET-23 EGFR signaling. Based on our genetic and biochemical data, we propose that ERM-1 binds to and sequesters the LET-23 EGFR in an inactive compartment at or close to the basolateral membrane of the VPCs. In this manner, ERM-1 competes with the activating LET-23/LIN-2/LIN-7/LIN-10 complex [5]. ERM-1 may act as a buffer that prevents the immediate activation of the entire pool of basolateral LET-23 EGFR at vulval induction and thus allows the generation of a prolonged signal.

## Results

### An In Vivo Screen Identifies Novel Regulators of LET-23 EGFR Localization and Expression

We performed RNAi knock-down of all genes (705 clones) reported to exhibit a protruding vulva (Pvl) phenotype, which is indicative of a defect in vulval fate specification or execution (**Table S1**) and examined LET-23 localization and expression in the vulval epithelium of live L3 larvae expressing a functional LET-23::GFP reporter (**Figure 1C–E**). The LET-23::GFP reporter used showed the same vulval expression pattern as endogenous LET-23 detected by antibody staining [5], and LET-23::GFP protein levels in total extracts were comparable to endogenous LET-23 levels (**Figure S1A**). Moreover, *let-23::gfp* efficiently rescued the *let-23(sy1)* vulvaless (Vul) phenotype (**Figure S1B**), and RNAi against *lin-7* or a mutation in *lin-2* caused an apical mislocalization of LET-23::GFP, as shown previously for endogenous LET-23 by antibody staining [5] (**Figure 1I**, and **Figure S1D**). In total, we identified 81 candidates that change different aspects of LET-23::GFP expression or localization (**Table 1**). We further classified these genes according to the specific mislocalization phenotypes observed (**Figure 1F**): Apical enrichment (24 genes, **Figure 1I,J**), accumulation in intracellular punctae or uniform cytoplasmic distribution (23 genes, **Figure 1M** and **Figure S2,C,D**), persisting expression in the 2° cells (31 genes, **Figure 1K** **and Figure S2E**), enrichment on the lateral membrane (2 genes, **Figure S2F**) and complex mislocalization phenotypes (10 genes, **Figure 1L**). Grouping the 81 genes into Clusters of Orthologous Groups (KOGs) indicated that a variety of processes are involved in regulating LET-23 localization (**Figure 1G**) [12]. In particular, genes involved in transcription, intracellular trafficking, signal transduction and protein stability and posttranslational modification were slightly overrepresented, while genes involved in chromatin modification, DNA replication and repair were underrepresented when compared to the distribution of the KOGs among the genes causing a Pvl phenotype that were screened.

**Table 1:** Genes that control the localization or expression of LET-23::GFP.

Gene	KOG Information	LET-23 localization	Known functions in vulval development
	***RNA processing and modification***		
*C18A3.3*	Nucleolar protein-like/EBNA1-binding protein	Punctate	
*rsp-6*	Alternative splicing factor SRp20/9G8 (RRM superfamily)	Persistent in 2°	
*prp-4*	U4/U6 small nuclear ribonucleoprotein Prp4 (WD40 repeats)	Other complex	
*ddx-23*	U5 snRNP-like RNA helicase subunit	Persistent in 2°	
*npp-17*	mRNA export protein (contains WD40 repeats)	Persistent in 2°	
*prpf-4*	U4/U6-associated splicing factor PRP4	Other complex	
*aly-2*	RRM motif-containing protein	Apical	
*ccf-1*	mRNA deadenylase subunit	Persistent in 2°	
*larp-1*	La RNA-binding motif	Persistent in 2°	Likely affects oogenesis via regulation of Ras-MAPK signaling
	***Chromatin Structure and dynamics***		
*lin-9*	Retinoblastoma pathway protein LIN-9/chromatin-associated protein Aly	Other complex	Negative regulation of the RTK/Ras-mediated signal transduction pathway that controls vulval development
*met-2*	Histone methyltransferase	Apical	Negatively regulates lin-3 transcription to restrict vulval development to three of the six VPCs
	***Energy production and conversion***		
*nduf-7*	NADH-ubiquinone oxidoreductase, NUFS7/PSST	Apical	
*aco-2*	Aconitase/homoaconitase (aconitase superfamily)	Persistent in 2°	
	***Cell cycle control and mitosis***		
*air-2*	Serine/threonine protein kinase	Punctate	
*ned-8*	Ubiquitin-like protein	Cytoplasmic	
*cdc-37*	Cell division cycle 37 protein, CDC37	Cytoplasmic/Persistent in 2°	
*mat-1*	DNA-binding cell division cycle control protein	Lateral	
	***Translation***		
*eif-3.E*	Translation initiation factor 3, subunit e (eIF-3e)	Punctate	
*C30C11.1*	Mitochondrial ribosomal protein L32	Apical	
*fbf-2*	Translational repressor Pumilio/PUF3 and related RNA-binding proteins	Persistent in 2°	Inhibits primary vulval cell fate specification
*iftb-1*	Translation initiation factor 2, beta subunit (eIF-2beta)	Punctate	
*alg-2*	Translation initiation factor 2C (eIF-2C) and related proteins	Apical/Cytoplasmic	
*T13H5.5*	Mitochondrial ribosomal protein S18b	Apical	
	***Transcription***		
*pbrm-1*	Chromatin remodeling complex RSC, subunit RSC1/Polybromo and related	Punctate	Interacts with two or more components of the EGF/RAS signaling pathway during vulval development
*lin-1*	Predicted transcription factor	Other complex	General effector of MAP kinase-mediated signaling required for vulval induction
*rpc-1*	RNA polymerase III, large subunit	Persistent in 2°/Apical	
*C55A6.9*	Putative RNA polymerase II regulator	Apical	
*let-381*	Transcription factor of the Forkhead/HNF3 family	Punctate	
*pax-3*	Homeodomain like	Persistent in 2°/Apical	
*lin-31*	Forkhead/HNF-3-related transcription factor	Persistent in 2°/complex	Tissue-specific effector of MAP kinase-mediated signaling in the vulva
*kin-10*	Casein kinase II, beta subunit	Persistent in 2°	
*T02C12.2*	Small nuclear RNA activating protein complex - 50kD subunit (SNAP50)	Persistent in 2°	
*rpb-11*	RNA polymerase, subunit L	Persistent in 2°	
*let-49*	Transcriptional coactivator	Other complex	
*tag-246*	SWI/SNF transcription activation complex subunit	Persistent in 2°	Required for full levels of LIN-3/EGF signaling during vulval development
*sptf-3*	Zn finger protein	Other complex	
	***Replication and repair***		
*hsr-9*	DNA damage checkpoint protein RHP9/CRB2/53BP1	Apical/Cytoplasmic	
	***Post-translational modification, protein turnover, chaperones***		
*tag-170*	Thioredoxin domain-containing	Apical	
*C11H1.3*	Predicted E3 ubiquitin ligase	Other complex	
*gsto-1*	Glutathione S-transferase	Apical	
*mig-6*	Serine proteinase inhibitor (KU family) with thrombospondin repeats	Cytoplasmic	
*usp-48*	Ubiquitin carboxyl-terminal hydrolase	Punctate	
*sig-7*	Predicted peptidyl prolyl cis-trans isomerase	Persistent in 2°/Apical	
*let-70*	Ubiquitin-protein ligase	Persistent in 2°	
*fnta-1*	Farnesyltransferase, alpha subunit/protein geranylgeranyltransferase type I	Punctate	
*rfp-1*	E3 ubiquitin ligase involved in syntaxin degradation	Apical	
	***Inorganic ion transport and metabolism***		
*tat-5*	P-type ATPase	Persistent in 2°	
	***General Functional Prediction only***		
*C06E4.6*	Reductases with broad range of substrate specificities	Persistent in 2°/Apical	
*ref-2*	Zn-finger	Apical	
*gex-3*	Membrane-associated hematopoietic protein	Persistent in 2°	
*F39B2.1*	Zn finger protein	Other complex	
*hrp-1*	RRM domain	Punctate/Persistent in 2°	
*hmg-1.2*	HMG box-containing protein	Apical	
*cdc-42*	Ras-related small GTPase, Rho type	Persistent in 2°	
*ngp-1*	Nucleolar GTPase	Apical	
*chp-1*	CHORD domain Co-chaperone	Cytoplasmic	
*ego-2*	Predicted signal transduction protein	Cytoplasmic	Positively regulates LIN-12/Notch signaling in the anchor cell-ventral uterine (AC/VU) cell fate decision
	***Function Unknown***		
*B0495.6*	Uncharacterized conserved protein	Cytoplasmic	
*F27C1.6*	Uncharacterized conserved protein	Cytoplasmic	
*ZK265.6*	Uncharacterized conserved protein	Apical	
*nsh-1*	Conserved nuclear protein	Cytoplasmic	Strawberry notch homolog, positively regulates *lin-3/egf* expression during RAS-dependent vulval induction
*F43D2.1*	G1/S-specific cyclin C like	Other complex	
*cdt-2*	WD domain g-beta repeat	Persistent in 2°	
*K12H4.5*	Unknown	Apical	
	***Signal Transduction***		
*abi-1*	Abl interactor ABI-1, contains SH3 domain	Persistent in 2°	
*sel-8*	Nuclear protein glutamine/asparagine (Q/N)-rich (‘prion’) domain	Persistent in 2°	Required for GLP-1 and LIN-12 signaling
*mpk-1*	Mitogen-activated protein kinase	Apical	Mitogen-activated protein (MAP) kinase ERK ortholog required for vulval cell fate specification
*nud-1*	Nuclear distribution protein NUDC	Apical	
*par-3*	PDZ protein	Persistent in 2°	
*par-2*	RING finger	Persistent in 2°	
*cki-1*	Cyclin-dependent kinase inhibitor	Cytoplasmic	
*sys-1*	Armadillo Repeats	Persistent in 2°	Functions in a Wnt/MAPK signaling pathway as a beta-catenin-like transcriptional coactivator
*ppfr-4*	Protein phosphatase 2A-associated protein	Lateral	
*pry-1*	Member of the Axin family of proteins	Persistent in 2°	Negative regulator of Wnt signaling pathways
	***Intracellular trafficking and secretion***		
*sft-4*	Putative cargo transport protein ERV29	Persistent in 2°	
*arf-3*	GTP-binding ADP-ribosylation factor Arf1	Cytoplasmic	
*sel-9*	Transmembrane emp24 domain protein	Apical	Likely functions to negatively regulate the transport of LIN-12 and GLP-1 to the cell surface
*aex-6*	GTPase Rab27, small G protein superfamily	Persistent in 2°	
	***Nuclear structure***		
*npp-11*	Nuclear pore complex, Nup98 component (sc Nup145/Nup100/Nup116)	Punctate	
	***Cytoskeleton***		
*erm-1*	Radixin, moesin and related proteins of the ERM family	Apical	
*wve-1*	Wiskott Aldrich syndrome proteins	Punctate	

### Changes in LET-23 EGFR Localization Alter RAS-Mediated Signaling

For a subset of the candidates with predicted roles in signaling or trafficking, we examined whether inhibition of these genes altered the activity of the RAS/MAPK pathway. This was tested by performing RNAi in the sensitized *let-60 ras(n1046)* gain-of-function background in which more than 3 VPCs are induced [13] and scoring the average number of induced VPCs per animal (**Figure 1Q**). It should be noted that the VPCs in the *let-60 ras(n1046)* gain-of-function background are still sensitive to the AC signal [13]. In those cases where RNAi caused a penetrant embryonic or larval lethal phenotype, we performed Pn.p cell-specific RNAi using an *rde-1(ne219lf); let-60(n1046gf)* RNAi resistant background expressing *rde-1(wt)* from the Pn.p cell-specific *lin-31* promoter [14]. For example, RNAi against *sft-4* or against the small GTPase *aex-6* caused persistent LET-23::GFP expression in 2° VPCs (**Figure 1K** and **Table 1**). Moreover, Pn.p cell-specific *sft-4* RNAi significantly enhanced vulval induction in the *let-60(gf)* background (**Figure 1Q**). The yeast *sft-4* homolog ERV29 encodes a SURF protein with a putative di-lysine endoplasmic reticulum (ER) localization signal that sorts secretory cargo proteins in the ER into COPII vesicles [15]. An *sft-4::gfp* translational reporter showed expression in the VPCs in perinuclear structures that resemble the ER (**Figure 1N**). Thus, ER to Golgi transport might be involved in controlling LET-23 turnover in the VPCs.

RNAi of *C11H1.3* caused a complex mislocalization phenotype with a moderate apical enrichment, punctate LET-23::GFP accumulation at or close to the apical membrane (**Figure 1L**) and an increase in vulval induction in the *let-60(n1046gf)* background (**Figure 1Q**). C11H1.3 encodes a predicted E3 ubiquitin ligase that is expressed in the VPCs in intracellular vesicles (**Figure 1O**). Therefore, C11H1.3 may control LET-23 localization and stability through ubiquitination of the receptor itself or of an associated factor. A penetrant mislocalization phenotype with punctate cytoplasmic accumulation of LET-23::GFP was observed in *ego-2* RNAi treated animals (**Figure 1M**), and a translational *ego-2::gfp* reporter was expressed in the cytoplasm and nuclei of all the VPCs (**Figure 1P**). *ego-2* encodes a BRO1 domain protein that is related to mammalian PTPN23, which regulates the transport of ubiquitinated EGFR through the ESCRT III complex to the intralumenal vesicles of multivesicular bodies [16]. Interestingly, *ego-2* has also been reported to regulate GLP-1 NOTCH signaling during germ cell development and embryogenesis as well as LIN-12 NOTCH signaling during somatic gonad development [17]. Therefore, *ego-2* might be a general regulator of LET-23 EGFR and LIN-12/GLP-1 NOTCH via control of their endocytic transport.

### ERM-1 Controls LET-23 EGFR Trafficking

**Figure 2:**
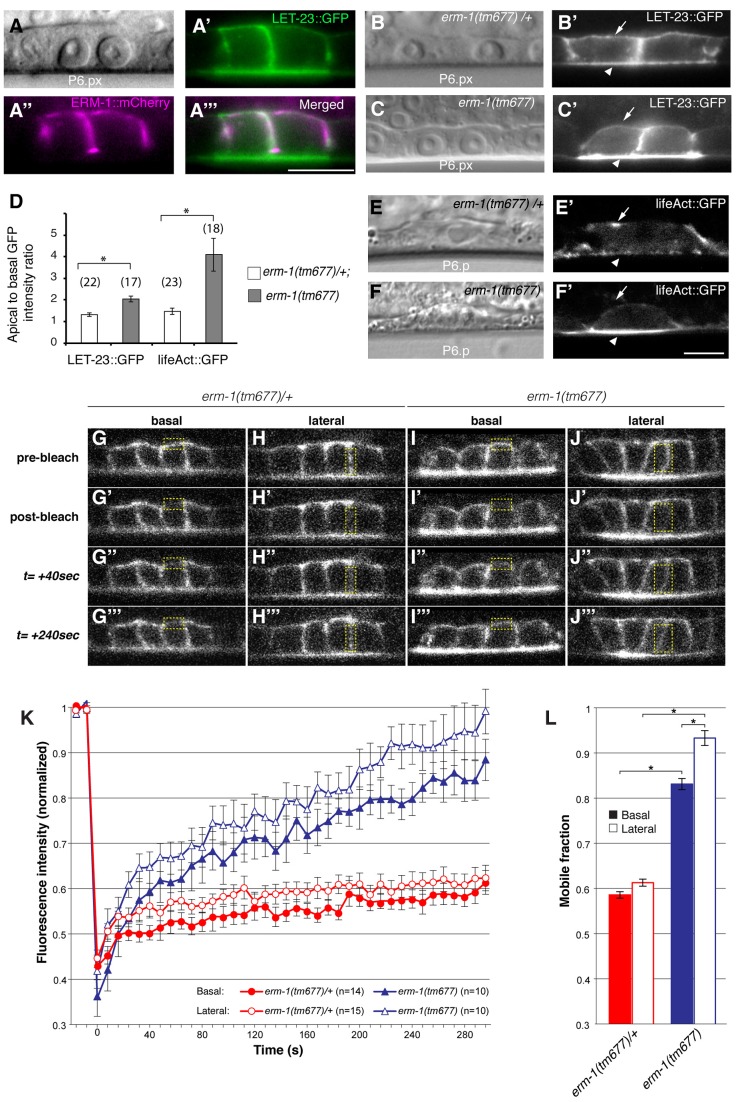
ERM-1 controls LET-23::GFP localization at the basolateral membrane of the vulval cells. (A) Nomarski image, (A′) LET-23::GFP (green) and (A″) ERM-1::mCherry (magenta) expression at the Pn.px stage. (A′″) shows a merged image of (A′) and (A″) indicating partial co-localization at the basolateral membrane. (B) Nomarski and (B′) LET-23::GFP expression in a heterozygous *erm-1(tm677)/+* and (C, C′) a homozygous *erm-1(tm677)* larva at the Pn.px stage. Arrows indicate the basal and arrowheads the apical membrane domains. (D) Apical to basal LET-23::GFP and lifeAct::GFP intensity ratios in P6.p in *erm-1(tm677)/+* versus *erm-1(tm677)*. The numbers of animals analyzed are indicated in brackets. Error bars represent the standard error of the mean. (E) Nomarski and (E′) lifeAct::GFP expression in P6.p of a heterozygous *erm-1(tm677)/+* and (F, F′) a homozygous *erm-1(tm677)* larva. (G–J′″) Example images of the FRAP experiment at the time points indicated to the left of panels G-G′″. (G-G′″ and I-I′″) Basal and (H-H′″ and J-J′″) lateral membrane regions outlined with the dotted yellow boxes were photobleached in heterozygous *erm-1(tm677)/+* and homozygous *erm-1(tm677)* larvae, respectively, at the Pn.pxxx stage. (K) Quantification of the FRAP experiments. The y axis indicates LET-23::GFP intensity normalized to the signal intensity measured before bleaching inside the bleached areas and to the total signal intensity in the cell, and the x-axis the time after photo-bleaching. The numbers of animals analyzed are shown in brackets. (L) Quantification of the mobile fraction from the FRAP curves. *Indicates p<0.05, as determined in a two tailed student's t-test - two-sample unequal variance. The scale bars are 10 µm.

One of the strongest apical enrichment mislocalization phenotypes was observed in *erm-1* RNAi treated animals (**Figure 1J**), prompting us to analyze the role of ERM-1 in LET-23 localization and signaling in more detail. *erm-1* encodes the sole *C. elegans* member of the Ezrin, Radixin and Moesin (ERM) protein family. ERM proteins link the cortical actin cytoskeleton and the plasma membrane and recruit transmembrane proteins to specific membrane compartment [18]. In addition, *C. elegans* ERM-1 is required for apical lumen morphogenesis in the intestine [19] [20]. In contrast to the apical localization observed in the intestine, an ERM-1::mCherry reporter showed basolateral and junctional localization and a partial overlap with LET-23::GFP in the VPCs and their descendants (**Figure 2A-A″**). Only after vulval invagination (at the Pn.pxxx stage), ERM-1 relocalized to the apical, luminal plasma membrane of the vulval toroids (data not shown). To confirm the RNAi phenotype, we examined LET-23::GFP expression in *erm-1(tm677)* null mutants. Homozygous *erm-1(tm677)* larvae showed decreased basolateral and increased apical membrane localization of LET-23::GFP in the VPCs and their descendants, resulting in a significantly increased ratio of apical to basolateral LET-23::GFP signal intensity when compared to heterozygous *erm-1(tm677)/+* controls (**Figure 2B–D**). The localization of the apical junction marker DLG-1::RFP [21] or the plasma membrane marker CED-10::GFP [22] were not changed, indicating that overall polarity of the VPCs was not altered in *erm-1(tm677)* mutants (data not shown and **Figure S3**). However, we detected a reduced basolateral staining of the F-actin reporter lifeAct::GFP [23] in the VPCs of *erm-1(tm677)* mutants, which is consistent with the role of ERM proteins as membrane linkers for cortical F-actin (**Figure 2D–F′**).

To test whether the reduced basolateral expression of LET-23::GFP is due to decreased basolateral secretion or to an increased membrane mobility and recycling rate of LET-23, we performed Fluorescence Recovery After Photobleaching (FRAP) experiments on the basal and lateral membranes of the vulval cells at the Pn.pxx stage and calculated the mobile fraction and half time of recovery (t_1/2_) of LET-23::GFP (**Figure 2G–L**) (see materials and methods). In *erm-1(tm677)* larvae, the total mobile fraction of LET-23::GFP was significantly higher than in heterozygous controls in both the basal and lateral compartments, while the t_1/2_ was not significantly changed (**Figure 2L**, t_1/2_ = 76 sec in heterozygous *erm-1(tm677)/+* vs. 81 sec in homozygous *erm-1(tm677)* mutants). Thus, *erm-1(tm677)* mutants exhibit an increased mobility of LET-23::GFP on the basolateral plasma membrane, rather than a decreased rate of basolateral secretion or retention.

### ERM-1 Inhibits Ligand-Dependent Internalization of LET-23 EGFR

**Figure 3:**
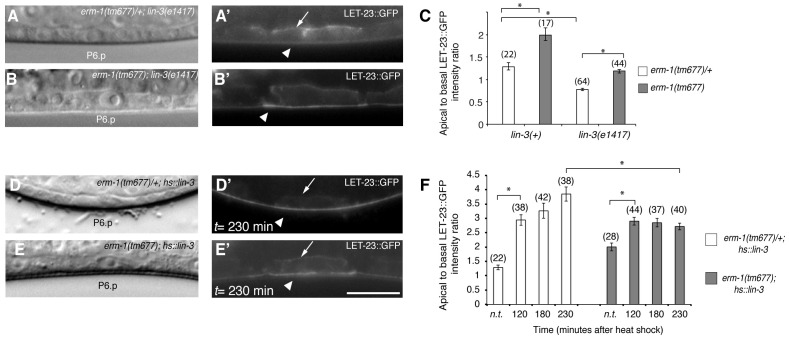
The LIN-3 EGF ligand stimulates and ERM-1 inhibits internalization and recycling of LET-23 on the basolateral membrane. (A) Nomarski image and (A′) LET-23::GFP expression in *erm-1(tm677)/+; lin-3(e1417)* and (B, B′) *erm-1(tm677); lin-3(e1417)* double mutants at the Pn.p stage. Arrows indicate the basal and arrowheads the apical membrane domains. (C) Apical to basal LET-23::GFP intensity ratios in P6.p in *erm-1(tm677)/+* versus *erm-1(tm677)* single and in *erm-1(tm677)/+; lin-3(e1417)* versus *erm-1(tm677); lin-3(e1417)* double mutants. (D) Nomarski image and (D′) LET-23::GFP expression in a heterozygous *erm-1(tm677)/+* and (E,E′) a homozygous *erm-1(tm677)* mutant 230 minutes after heat-shock induction of LIN-3. (F) Apical to basal LET-23::GFP intensity ratios at different time points after heat-shock. n.t. indicates animals of the same genotype that were not subjected to a heat-shock. *Indicates p<0.001 as determined in a two tailed student's t-test - two-sample unequal variance. The scale bars are 10 µm.

Changes in the ligand concentrations could alter the steady-state levels of LET-23 EGFR on the basolateral membrane. For example, reducing the dose of LIN-3 EGF may decrease receptor endocytosis and thus diminish the ratio of apical to basal EGFR, while increasing the dose of LIN-3 may promote receptor endocytosis on the basolateral membrane and therefore increase the apical to basal ratio. On the other hand, mutations in components of the LIN-2/LIN-7/LIN-10 complex that is necessary to retain the EGFR on the basolateral membrane also cause a strong reduction in basolateral EGFR localization, yet they result in reduced receptor activation [5]. To distinguish between these different scenarios, we tested if the increased apical LET-23::GFP localization in *erm-1(tm677)* mutants could be due to a higher rate of LET-23 endocytosis after binding to LIN-3 EGF secreted from the AC. In *lin-3(e1417)* mutants, in which LIN-3 activity in the AC is strongly reduced [24], apical LET-23::GFP localization was nearly two-fold reduced (**Figure 3A,C**). However, in *erm-1(tm677)*; *lin-3(e1417)* double mutants the apical to basal LET-23::GFP ratio was lower than in *erm-1(tm677)* but higher than in *lin-3(e1417)* single mutants (**Figure 3B,C**). Since the viable *lin-3(e1417)* allele used does not eliminate all LIN-3 activity, we conclude that the apical accumulation of LET-23::GFP in the absence of ERM-1 is at least in part ligand-dependent. On the other hand, a pulse of ectopic LIN-3 ubiquitously expressed under control of the heat-shock promoter *hs*::*lin-3* [25] caused the almost complete disappearance of LET-23::GFP from the basolateral membrane and accumulation on the apical membrane within 230 minutes (**Figure 3D,F**). In homozygous *erm-1(tm677)* mutants, however, a LIN-3 pulse caused a smaller increase in the apical LET-23::GFP pool and persisting receptor expression on the basolateral membrane (**Figure 3E,F**). Thus, not only LET-23 endocytosis but also basolateral recycling are increased in *erm-1(tm677)* mutants, which is consistent with the increased mobile fraction of LET-23::GFP observed in the FRAP experiments (**Figure 2L**). By contrast, activation of the EGFR signaling pathway downstream of the receptor using for example the *let-60(gf)* mutation did not change LET-23::GFP localization (data not shown). Thus, the LIN-3 ligand stimulates and ERM-1 inhibits internalization and recycling of LET-23 on the basolateral membrane.

### ERM-1 Acts as a Negative Regulator of the EGFR/RAS/MAPK Pathway

**Figure 4:**
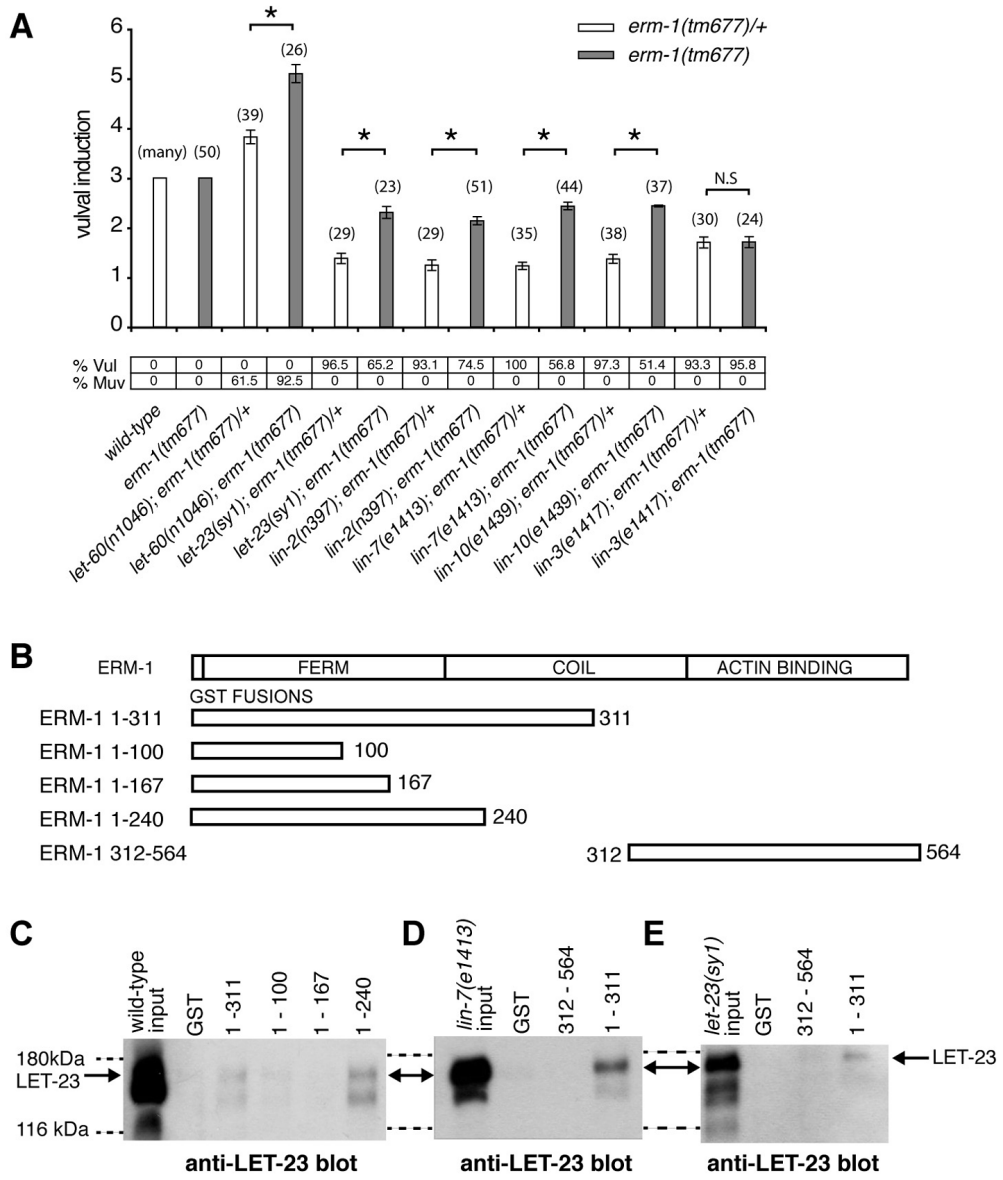
ERM-1 negatively regulates vulval induction and binds to LET-23. (A) Genetic epistasis analysis between *erm-1* and components of the *egfr/ras/mapk* pathway. Vulval induction (VI) indicates the average numbers of induced VPCs in different double mutant combinations scored in *erm-1(tm677)* heterozygous (white bars) versus homozygous (gray bars) animals. %Vul indicates the fraction of animals with VI<3 and % Muv the fraction of animals with VI>3. The numbers of animals scored for each genotype are indicated in brackets. N.S: no significant change. *Indicates p<0.05 as determined in a two tailed student's t-test - two-sample unequal variance. (B) Structures of the GST::ERM-1 fusion proteins tested for LET-23 binding. (C) Interaction of LET-23 from wild-type extracts with different GST::ERM-1 fusion proteins detected on an anti-LET-23 Western blot. (D) Binding of LET-23 extracted from *lin-7(e1413)* and (E) from *let-23(sy1)* mutants to GST::ERM-1 proteins. The dashed lines indicate the approximate positions of the 180 kDa and 116 kDA protein standards.

Enhanced receptor endocytosis could result in the attenuation of LET-23 signaling, while faster recycling to the plasma membrane could promote signaling [1]. To determine how the altered LET-23 dynamics in *erm-1* mutants affects signaling, we performed epistasis analysis by combining *erm-1(tm677)* with mutations in different components of the EGFR/RAS/MAPK pathway [4] and quantifying vulval induction. In *erm-1(tm677)* single mutants, the three proximal VPCs were always induced as in the wild-type (**Figure 4A**). However, in *let-60(gf); erm-1(tm677)* double mutants, the average number of induced VPCs was significantly increased compared to *let-60(gf); erm-1(tm677)/+* controls, resulting in an enhanced Multivulva (Muv) phenotype (**Figure 4A**). Thus, ERM-1 negatively regulates RAS/MAPK signaling during vulval induction. Mutations in the *lin-2/lin-7/lin-10* receptor localization complex or in the PDZ binding motif in *let-23*(*sy1*) cause a penetrant Vulvaless (Vul) phenotype because LET-23 mislocalized to the apical membrane cannot bind to LIN-3 [5]. Interestingly, *erm-1(tm677)* partially suppressed the *lin-2(n397)*, *lin-7(e1413)*, *lin-10(e1439)* and *let-23(sy1)* Vul phenotypes (**Figure 4A**), indicating that ERM-1 inhibits vulval induction independently of the LIN-2/LIN-7/LIN-10 receptor localization complex. However, the suppression of the *lin-2(n397)* Vul phenotype was not accompanied by a visible re-localization of LET-23::GFP to the basolateral membrane (data not shown). In contrast, *erm-1(tm677)* did not suppress the *lin-3(e1417)* Vul phenotype, suggesting that vulval induction in *erm-1(tm677)* mutants still depends on the AC signal (**Figure 4A**).

### ERM-1 Forms a Complex with LET-23 EGFR Independently of the LIN-2/LIN-7/LIN-10 Complex

ERM proteins are composed of an N-terminal FERM domain and a C-terminal actin-binding domain [26]. They can switch from a closed, inactive conformation in the cytoplasm to an open, active conformation at the plasma membrane [27]. The FERM domain in the open conformation interacts with plasma membrane proteins either directly or indirectly through adaptor proteins [26], while binding of the actin cytoskeleton to the C-terminus of ERM proteins regulates the activity of the entire complex [28]. Since our genetic analysis indicated that ERM-1 controls LET-23 signaling independently of the LIN-2/LIN-7/LIN-10 complex, we tested if ERM-1 and LET-23 might exist in an alternate complex. For this purpose, different portions of purified recombinant ERM-1 tagged at the N-terminus with glutathione S-transferase (GST) were incubated with total worm lysates, and bound LET-23 was detected on Western-blots. LET-23 from wild-type worm extracts bound to N-terminal fragments containing the entire FERM domain (GST::ERM-1_1–240_ and GST::ERM-1_1–311_), while a C-terminal fragment (GST::ERM-1_312–564_) or truncated FERM domains (GST::ERM-1_1–100_ or GST::ERM-1_1–167_) did not bind LET-23 (**Figure 4B,C**). Moreover, LET-23 extracted from *sy1* mutants, in which LET-23 lacks the PDZ binding motif, or from *lin-7(e1413)* mutants still bound to the ERM-1 FERM domain (**Figure 4D,E**). Thus, LET-23 and ERM-1 exist in a complex that is distinct from the LIN-2/LIN-7/LIN-10 localization complex.

### ERM-1 Is a Temporal Regulator of EGFR Signaling

**Figure 5:**
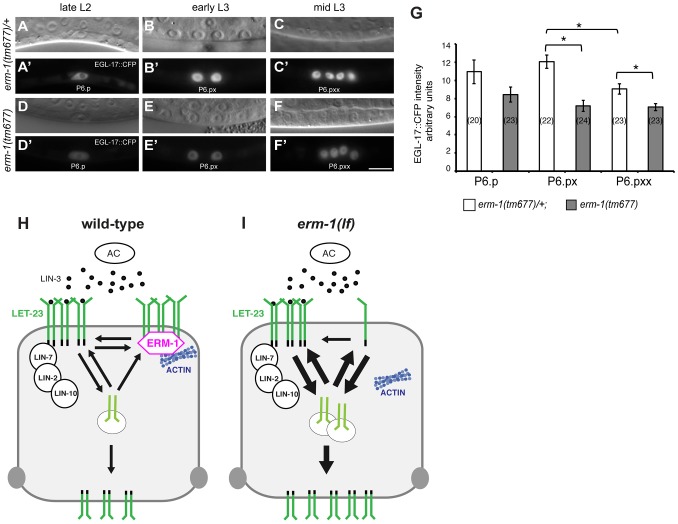
Temporal regulation of LET-23 EGFR signaling by ERM-1. (A, B, C) Nomarski images and (A′, B′, C′) EGL-17::CFP expression in *erm-1(tm677)/+* controls versus (D through F′) homozygous *erm-1(tm677)* mutants at the Pn.p (A′, D′), Pn.px (B′, E′) and Pn.pxx (C′, F′) stages. The scale bar is 10 µm. (G) Quantification of nuclear EGL-17::CFP intensities in P6.p and its descendants. The numbers of animals analyzed are indicated in brackets. *Indicates p<0.01 as determined in a two tailed student's t-test - two-sample unequal variance. Error bars represent the standard error of the mean. (H) Two antagonistic complexes control LET-23 localization at the basolateral membrane of the VPCs. The ternary LIN-2/LIN-7/LIN-10 complex promotes receptor activation, while the ERM-1 complex (magenta) prevents LET-23 endocytosis and signaling from basolateral membrane. ERM-1 may sequester LET-23 in an actin-rich membrane compartment to prevent receptor activation. (I) In the absence of ERM-1, more LET-23 can be activated via the LIN-2/LIN-7/LIN-10 complex, resulting in enhanced receptor endocytosis and recycling, and ultimately an increased accumulation of LET-23 on the apical plasma membrane.

The increased basolateral LET-23 mobility in *erm-1(tm677)* mutants may result in an overall elevated activity of the RAS/MAPK pathway, as more LET-23 molecules are available to interact with LIN-3. The co-localization of LET-23 and ERM-1 together with the *in vitro* protein interaction experiments suggested that both proteins form a complex at the basolateral membrane of the VPCs. We thus hypothesized that ERM-1 may prevent the instant activation of the entire basolateral pool of LET-23 once the AC begins to secrete LIN-3 at the mid L2 stage, allowing the cells to maintain a high LET-23 activity after vulval induction. To test this model, we quantified the expression levels of the RAS/MAPK target EGL-17::CFP [29] in the descendants of the VPCs. In wild-type mid L3 larvae, we observed a peak of EGL-17::CFP expression after vulval induction in the 1° descendants of P6.p (**Figure 5A–C′,G**). By contrast, *erm-1(tm677)* mutants showed a gradual decrease rather than an increase in EGL-17::CFP expression after vulval induction (**Figure 5D–G**). Thus, ERM-1 is required for the generation of a long-lasting RAS/MAPK signal in the 1° vulval cells after fate specification has occurred.

## Discussion

### Regulation of EGFR Localization Allows Pattern Formation

In order to systematically search for regulators of LET-23 EGFR trafficking and signaling, we performed an *in vivo* receptor localization screen in *C. elegans* larvae. There do exist certain limitations of this system, such as the inability to isolate individual cell for biochemical studies. However, an important advantage of our approach over previous studies performed with cultured epithelial cells [1] is the ability to observe the dynamics of receptor trafficking under normal conditions, in epithelial cells embedded in their natural environment and receiving physiological concentrations of various extracellular signals. The different regulators of LET-23 EGFR localization and signaling identified in our screen point at a complex network controlling LET-23 EGFR trafficking and signaling in different sub-cellular compartments. In a system, such as the VPCs, where ligand availability is limiting [8], [30], these additional control mechanisms at the level of the receptor are necessary to prevent too many cells from engaging in signaling at the same time and to focus the inductive signal on a single cell (P6.p). A perturbation of LET-23 EGFR trafficking can lead to a multivulva phenotype because decreased ligand sequestering by the 1° VPC P6.p results in increased LET-23 EGFR activation in the distal VPCs [30]. The down regulation of the LET-23 EGFR in all but the 1° VPC is therefore an important mechanism to break the symmetry of the initially equivalent VPCs and select a single cell for the 1° fate. The most frequent phenotype we observed in our screen (31 genes) was persisting LET-23 EGFR expression in 2° VPCs, and for those genes that had a significant effect on signaling we found increased rather than decreased vulval induction in the *let-60* background. This suggests that a relatively large number of negative regulators of EGFR signaling is required to generate the invariant pattern of vulval cell fates with a single 1° cell flanked by two 2° cells.

### Genes Regulating LET-23 EGFR Trafficking Are Conserved

A recent study has indicated that around 38% of all predicted protein coding genes in *C. elegans* possess at least one human homolog [31]. However, we found for 91% of the genes identified in our screen (74 of 81) at least one human homolog in the OrthoList, suggesting that the mechanisms regulating EGFR trafficking are strongly conserved. Further studies of these mammalian homologs may provide new means of interfering with deregulated EGFR signaling in human cells.

### ERM-1 Is a Temporal Regulator of EGFR Signaling

We describe a new function of the *C. elegans* Ezrin homolog ERM-1 in regulating EGFR signaling on the basolateral membrane of the vulval cells. Based on the subcellular localization and dynamics and on genetic and biochemical data, we propose that ERM-1 forms a complex with the LET-23 EGFR at the basolateral plasma membrane to recruit the receptor into an actin-rich inactive membrane compartment and limit receptor activation (**Figure 5H,I**). A similar function has been proposed for mammalian NF2 Merlin, which shares similarity to Ezrin/Radixin/Moesin proteins. In confluent cultured epithelial cells, Merlin coordinates adherens junction stabilization with EGFR signaling by recruiting the EGFR into an apical membrane compartment, where the receptor cannot be activated [32]. In analogy, ERM-1 may link a fraction of the LET-23 EGFR pool at the basolateral membrane to cortical F-actin and restrict the access of the receptor to the activating LIN-2/LIN-7/LIN-10 complex [5]. In the absence of the tripartite LIN-2/LIN-7/LIN-10 complex, most of the residual basolateral LET-23 EGFR is probably bound and inactivated by ERM-1. The inhibitory ERM-1 complex thus antagonizes the activating LIN-2/LIN-7/LIN-10 complex to prevent the instant activation and subsequent degradation of the entire basolateral pool of LET-23 EGFR once the AC begins to secrete LIN-3 in the mid-L2 stage. This mechanism allows the vulval cells to maintain high LET-23 EGFR activity at later time points after vulval induction. LET-23 EGFR may be released from the ERM-1 complex when the vulval lumen is formed and ERM-1 relocalizes to the apical plasma membrane of the toroids. Such a buffering mechanism may be important, as sustained RAS/MAPK signaling is required during the subsequent phase of vulval morphogenesis when RAS/MAPK activity induces the expression of genes required for the execution of the vulval fates [4], [33]. Thus, the strength and duration of EGFR activation during development must be precisely controlled to achieve the correct levels of RAS/MAPK activity required for organogenesis.

## Materials and Methods

### Strains and General Methods

*C. elegans* strains were maintained at 20°C on standard nematode growth media [34] and the reference wild-type strain of *C. elegans* used was Bristol N2. Mutant strains used: LGI: *erm-1(tm677)/hT2[bli-4(e937) let(q782) qIs48] (I;III), rde­1(ne219), lin-10(e1339)*. LGII: *let-23(sy1), lin-7(e1413), syIs12[hs::lin-3EGF]* [25]. LGIII: *unc-119(ed3), unc-119(e2498). LGIV: lin-3(e1417), let-60(n1046)*. LGX: *lin-2(n397)*. Integrated and extra-chromosomal arrays: *qyIs23[Pcdh-3::mCherry::plcδ^PH^; unc-119(+)] II* [35], *zhIs038[let-23::gfp, unc-119(+)] IV, zhEx484[C11H1.3::gfp; Pmyo-2::mCherry], zhIs396[Pdlg-1::lifeact::gfp::unc-54 3′utr, Plin-48::gfp]* [23], *zhEx486[sft-4::gfp; Pmyo-2::mCherry], zhEx487[ego-2::gfp; Pmyo-2::mCherry], zhEx519 [erm-1::mCherry; unc-119(+);Pmyo-2::mCherry]; zhEx418[Plin-31::rde-1; Pmyo-2::mcherry]*.

The construction of the translational reporter constructs used in this study is described in the **Text S1** and **Table S2** (see also **Figure S4**).

Extra-chromosomal arrays were obtained by microinjection of plasmids at 20 to 50 ng/µl along with the coinjection marker *Pmyo2::mCherry or unc-119(+)* at 2 to 10 ng/µl and pBluescript to a final concentration of 150 to 200 ng/µl as described [36]. *zhIs038* was obtained by bombardment of *unc-119* mutants with plasmid coated gold particles as described [37]. Primers used and details on the construction of plasmids can be found in the Supplementary Material.

### GST Pull-down and Western Blots

GST fusion proteins were purified from *E. coli* BL-21 with glutathione-sepharose beads, incubated with 500 µg total protein worm extracts, and bound LET-23 was detected on Western blots with affinity-purified rabbit anti-LET-23 antibodies (1∶1000 dilution) raised against the C-terminal 196 amino acids [5].

### RNAi Screen and Microscopy

RNAi was performed by bacterial feeding as described [38]. LET-23::GFP localization was scored in L3 larvae of the F1 generation mounted on 3% agarose pads supplemented with 5 mM tetramisol. For each RNAi clone, the vulval cells in 30 to 50 animals were observed at 40 to 63-fold magnification with a Leica DMRA wide-field microscope. Positive RNAi clones from the rescreen were verified by DNA sequencing. Images were recorded with a Hamamatsu ORCA-ER CCD camera controlled by the Openlab 5 software package (Improvision). Confocal images were recorded with a Olympus FV-1000 or a Zeiss LSM710 confocal microscope and analyzed with ImageJ [39]. Apical to basolateral intensity ratios were determined in mid-sagittal frames taken with the same illumination and same exposure settings by manually selecting the basal and apical membrane compartments and measuring total fluorescence intensities.

### Fluorescence Recovery after Photobleaching

Larvae at the Pn.pxx stage were imaged at 20°C using a 63×/1.4 NA oil lens on an Zeiss LSM710 confocal microscope equipped with 458/488/514 nm argon and 405 nm diode lasers. A selected area of the basal or lateral membrane was bleached using the 488 nm argon laser at 85% power setting for 886.6 µsec to bleach around 70% of the signal, and fluorescence recovery was monitored over the following 296 seconds taking a frame every 8 seconds with a 488 nm laser excitation at 20% power intensity, a pinhole equivalent to 2.12 Airey units, a frame size of 256×256 dpi, and a pixel size of 0.53 µm. Data were analyzed in ImageJ by first registering the images with the StackReg plugin and then using the FRAP Norm plugin by Joris Meys [40] to measure recovery. Normalized curves were fitted to the formula I(t) = A·(1-e^−kt^)+C using the solver function in MS Excel to calculate the total mobile fraction A and half time as t_1/2_ = 1/k.

### Heat Shock Treatment

Synchronized L3 larvae were heat-treated at 33°C for 30 minutes in a water bath, transferred to 20°C, and imaged at 120, 180, 230 minute after induction under the same illumination and exposure conditions. Quantification of the apical to basal LET-23::GFP intensity ratio with and without heat-shock treatment as described in the results.

## Supporting Information

Figure S1 Characterization of the LET-23::GFP reporter. (**A**) Expression of endogenous LET-23 (aprox. 150 kDa) and LET-23::GFP (aprox. 177 kD) in total worm lysates of L4 larvae and young adults detected on Western blots probed with anti-LET-23(left) and anti-GFP(right) antibodies. The upper band is LET-23::GFP and the lower band is endogenous LET-23. Integrated LET-23::GFP is expressed at similar levels as endogenous LET-23. (**B**) Complete rescue of the *let-23(sy1)* Vulvaless phenotype with an extra-chromosomal *let-23::gfp* array obtained by microinjection. The percentages of animals with a Vul phenotype in the presence an absence of the extra-chromosomal array are shown. The numbers of animals scored are indicated in brackets. (**C**) LET-23::GFP expressed from the integrated array used for the screen (*zhIs038*) is localized at basolateral and apical plasma membrane of the vulval cells in wild-type larvae, but (**D**) mislocalized to the apical compartment in *lin-2(n397)* mutants. (**D′**) shows the LET-23::GFP channel of (**D**) merged with the corresponding Nomarski picture. The scale bar is 10 µm.
(JPG)

Figure S2 Additional examples of genes identified in the receptor localization screen. In each row, the left panels show the Nomarski images and right panels the fluorescent images. (**A**) Normal LET-23::GFP expression in vector controls, (**B**) apical enrichment (arrow) in *ngp-1* RNAi, (**C**) punctate localization (arrow) in E04F6.4 RNAi, (**D**) cytoplasmic enrichment in *arf-3* RNAi, (**E**) persistent expression in 2° cells (asterisk) in *rab-27* RNAi, and (**F**) enrichment at the lateral membrane separating the two P6.p descendants in *ppfr-4* RNAi animals. The scale bars are 10 µm.
(JPG)

Figure S3 Polarity of the vulval cells in erm-1(tm677) mutants. (**A**) Nomarski image and (**A′**) Normal basolateral localization of the CED-10::GFP reporter in heterozygous *erm-1(tm677)/+* and (**B**) Nomarski image and (**B′**) localization of the CED-10::GFP reporter in homozygous *erm-1(tm677)* mutants. The scale bar is 10 µm.
(JPG)

Figure S4 Structure of (A) the LET-23::GFP and (B) ERM-1::mCherry reporter constructs.
(JPG)

Table S1 List of 705 Pvl genes used for the mislocalization/missexpression screen.
(DOCX)

Table S2 Sequences of primers used.
(DOCX)

Text S1 Details on the construction of the translational reporter constructs used in this study. A schematic drawing of the LET-23::GFP and ERM-1::mCherry constructs is shown in **Figure S4A**, and the sequences of primers used are listed in **Table S2**.
(DOC)
