# Supplementary figures and images for: An In Vivo EGF Receptor Localization Screen in C. elegans Identifies the Ezrin Homolog ERM-1 as a Temporal Regulator of Signaling
Source: PLoS Genet. 2014 May 1;10(5):e1004341. doi: 10.1371/journal.pgen.1004341 (PMC4006739; doi:10.1371/journal.pgen.1004341)

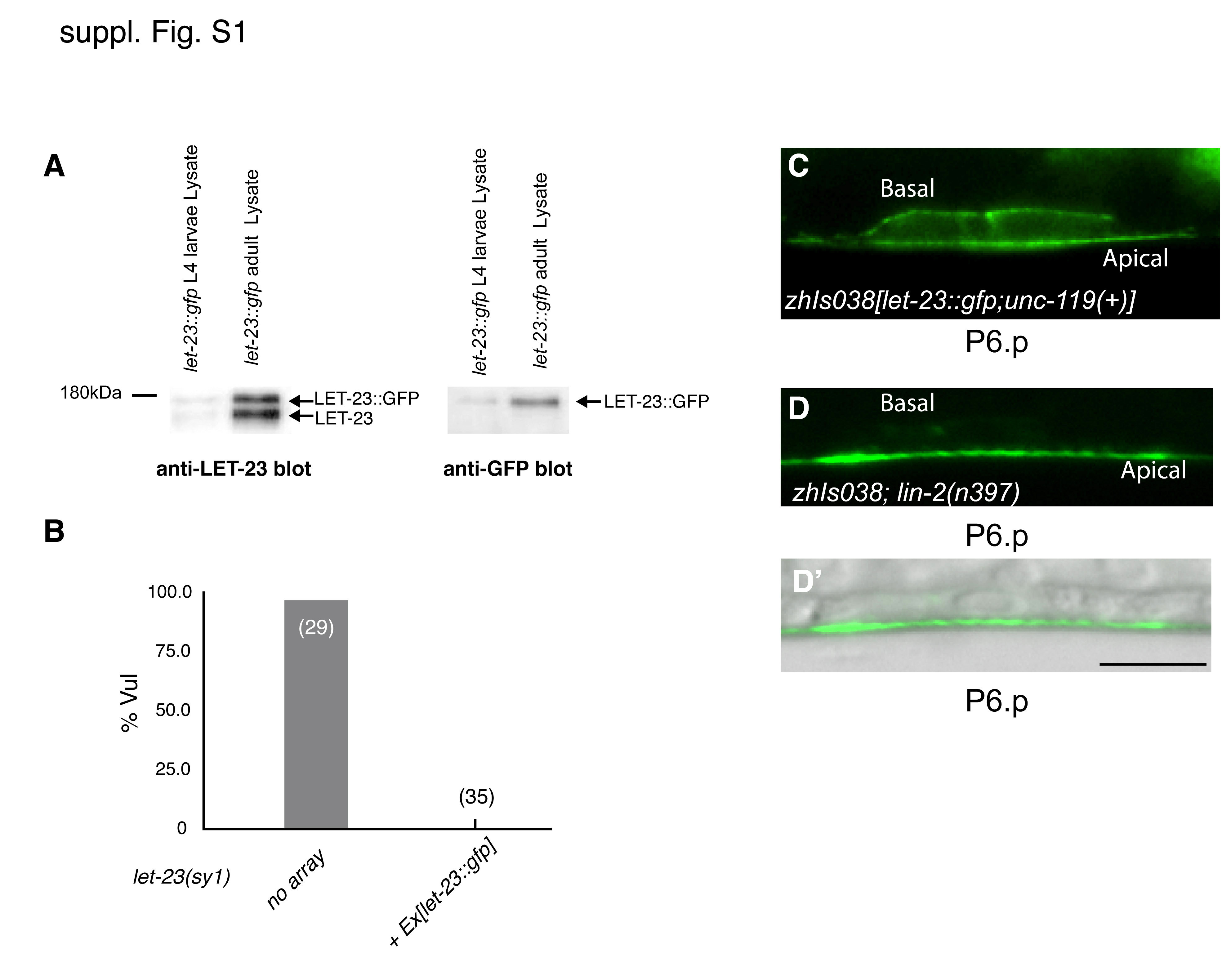

Supplement: Figure S1 — Characterization of the LET-23::GFP reporter. (A) Expression of endogenous LET-23 (aprox. 150 kDa) and LET-23::GFP (aprox. 177 kD) in total worm lysates of L4 larvae and young adults detected on Western blots probed with anti-LET-23(left) and anti-GFP(right) antibodies. The upper band is LET-23::GFP and the lower band is endogenous LET-23. Integrated LET-23::GFP is expressed at similar levels as endogenous LET-23. (B) Complete rescue of the let-23(sy1) Vulvaless phenotype with an extra-chromosomal let-23::gfp array obtained by microinjection. The percentages of animals with a Vul phenotype in the presence an absence of the extra-chromosomal array are shown. The numbers of animals scored are indicated in brackets. (C) LET-23::GFP expressed from the integrated array used for the screen (zhIs038) is localized at basolateral and apical plasma membrane of the vulval cells in wild-type larvae, but (D) mislocalized to the apical compartment in lin-2(n397) mutants. (D′) shows the LET-23::GFP channel of (D) merged with the corresponding Nomarski picture. The scale bar is 10 µm. (JPG) [file pgen.1004341.s001.jpg]

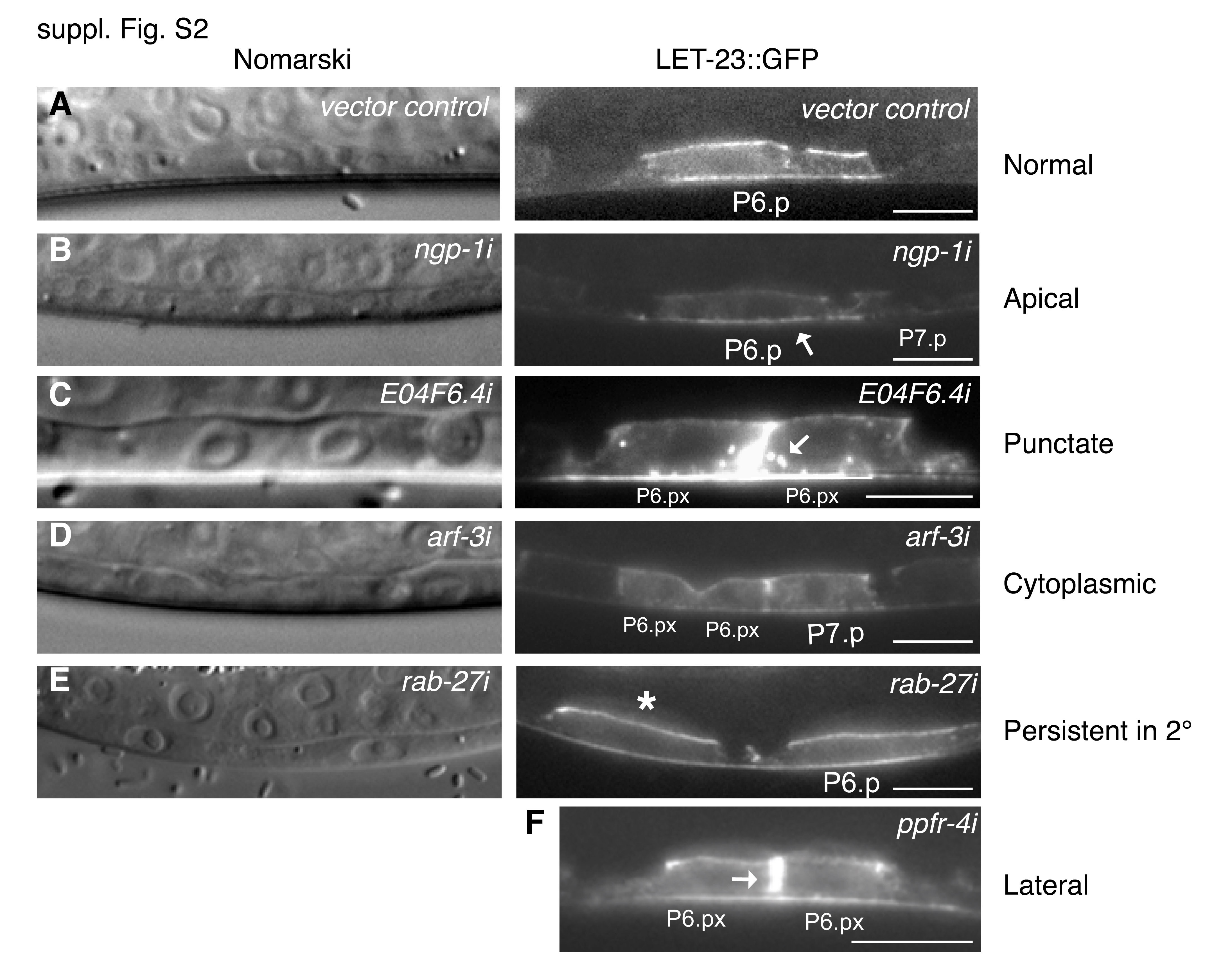

Supplement: Figure S2 — Additional examples of genes identified in the receptor localization screen. In each row, the left panels show the Nomarski images and right panels the fluorescent images. (A) Normal LET-23::GFP expression in vector controls, (B) apical enrichment (arrow) in ngp-1 RNAi, (C) punctate localization (arrow) in E04F6.4 RNAi, (D) cytoplasmic enrichment in arf-3 RNAi, (E) persistent expression in 2° cells (asterisk) in rab-27 RNAi, and (F) enrichment at the lateral membrane separating the two P6.p descendants in ppfr-4 RNAi animals. The scale bars are 10 µm. (JPG) [file pgen.1004341.s002.jpg]

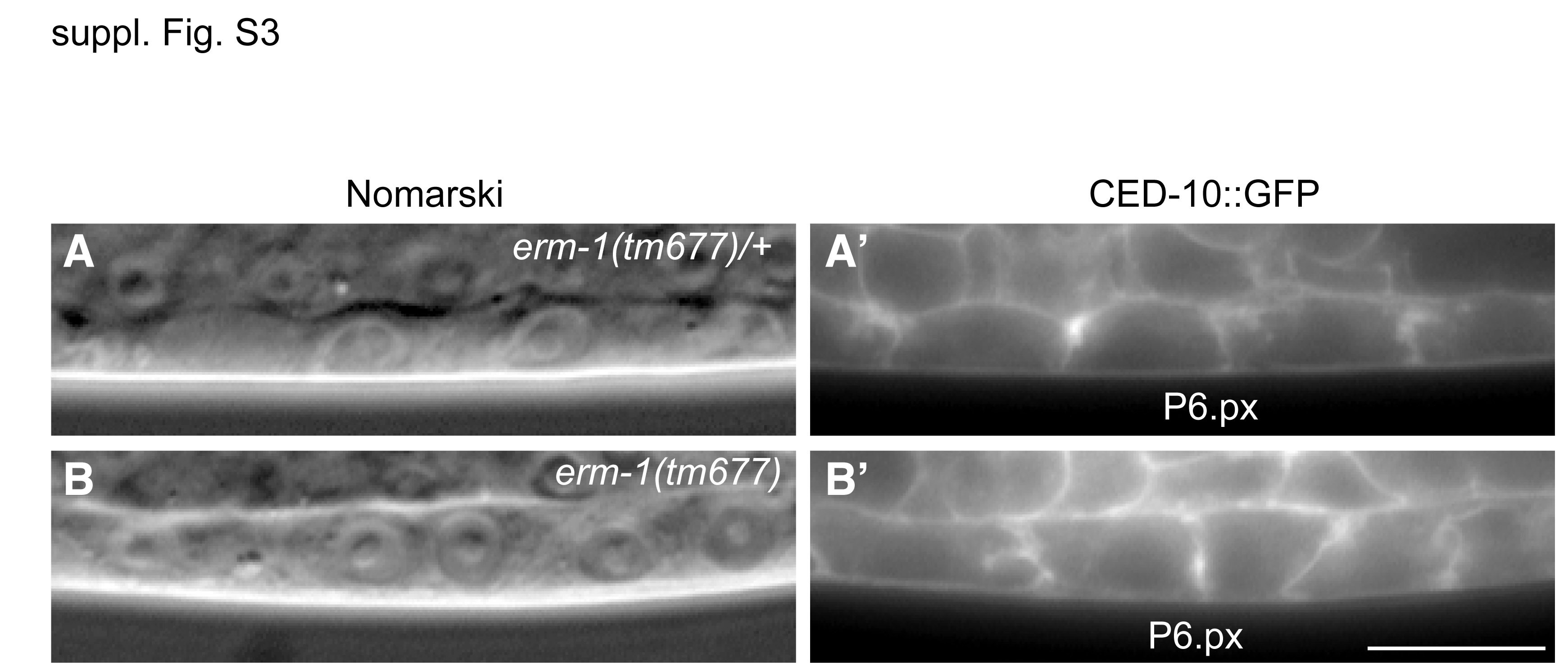

Supplement: Figure S3 — Polarity of the vulval cells in erm-1(tm677) mutants. (A) Nomarski image and (A′) Normal basolateral localization of the CED-10::GFP reporter in heterozygous erm-1(tm677)/+ and (B) Nomarski image and (B′) localization of the CED-10::GFP reporter in homozygous erm-1(tm677) mutants. The scale bar is 10 µm. (JPG) [file pgen.1004341.s003.jpg]

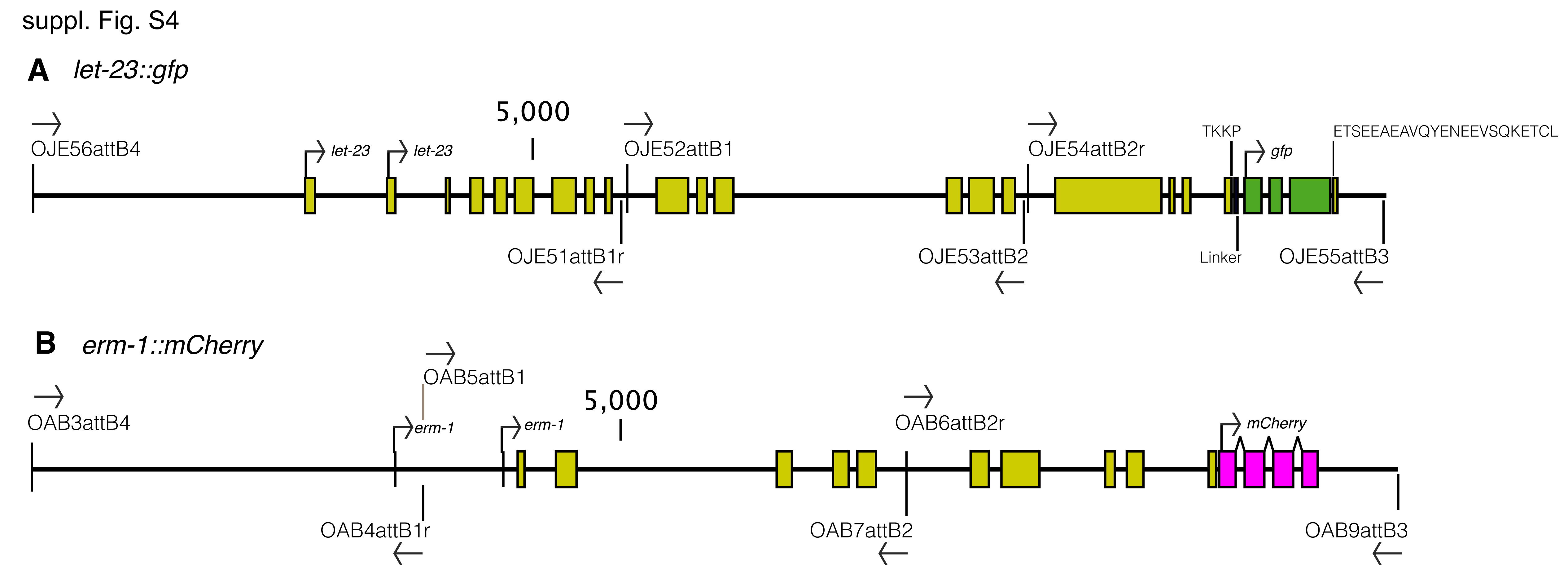

Supplement: Figure S4 — Structure of (A) the LET-23::GFP and (B) ERM-1::mCherry reporter constructs. (JPG) [file pgen.1004341.s004.jpg]
