# Supplementary material for: An In Vivo EGF Receptor Localization Screen in C. elegans Identifies the Ezrin Homolog ERM-1 as a Temporal Regulator of Signaling
Source: PLoS Genet. 2014 May 1;10(5):e1004341. doi: 10.1371/journal.pgen.1004341 (PMC4006739; doi:10.1371/journal.pgen.1004341)
Supplement: Table S1 — List of 705 Pvl genes used for the mislocalization/missexpression screen. (DOCX) [file pgen.1004341.s005.docx]

| Sequence name | Gene name | Description |
| --- | --- | --- |
| ZC123.3 | ZC123.3 | RNA-binding protein C2H2 Zn-finger domain |
| R06A10.2 | gsa-1 | G-protein alpha subunit group S |
| K12C11.2 | smo-1 | Ubiquitin domain |
| C53H9.2 | C53H9.2 |  |
| W10C8.2 | pop-1 |  |
| M01B12.5 | M01B12.5 | RIO1/ZK632.3/MJ0444 family |
| W01B11.3 | nol-5 | Putative snoRNA binding domain |
| C46H11.6 | C46H11.6 |  |
| W05F2.4 | W05F2.4 | Proline-rich region |
| T12F5.1 | T12F5.1 |  |
| Y8G1A.2 | inx-13 | Innexin |
| ZK770.3 | inx-12 | Innexin |
| F53F10.5 | npp-11 |  |
| T03F1.8 | T03F1.8 | Guanylate kinase |
| F55C7.7 | unc-73 | Dbl domain (dbl/cdc24 rhoGRF family) |
| C18E3.2 | C18E3.2 | BAF60b domain of the SWIB complex |
| C43E11.9 | C43E11.9 |  |
| C43E11.10 | cdc-6 | HMG-I and HMG-Y DNA-binding domain (A+T-hook) |
| M04F3.1 | rpa-2 |  |
| F28B3.7 | him-1 | ABC transporters family |
| W05F2.3 | W05F2.3 |  |
| F48C1.4 | F48C1.4 |  |
| F55A12.8 | F55A12.8 |  |
| C30H7.2 | C30H7.2 | Thioredoxin family |
| F27C1.6 | F27C1.6 |  |
| T05E8.3 | T05E8.3 | DEAD/DEAH box helicase |
| F55F8.2 | F55F8.2 | DEAD/DEAH box helicase |
| F55F8.4 | cir-1 |  |
| F55F8.5 | tag-345 | G-protein beta WD-40 repeats |
| C32F10.5 | hmg-3 | HMG1/2 (high mobility group) box |
| F33D11.10 | F33D11.10 | ATP-dependent helicase, DEAD-box |
| B0207.4 | air-2 | Eukaryotic protein kinase |
| C06A5.1 | C06A5.1 |  |
| B0207.6 | B0207.6 |  |
| C06A5.3 | C06A5.3 |  |
| T27A3.2 | T27A3.2 | Ubiquitin-associated domain |
| T09B4.1 | T09B4.1 |  |
| T08B2.8 | T08B2.8 |  |
| F26B1.7 | let-381 | Fork head domain |
| ZC308.1 | gld-2 | PAP/25A core domain |
| ZC328.4 | san-1 |  |
| T23H2.5 | rab-10 | Ras family |
| F37E3.1 | ncbp-1 | Cytochrome b/b6 |
| C55B7.5 | uri-1 |  |
| F57B10.8 | F57B10.8 |  |
| W02D3.9 | unc-37 | G-protein beta WD-40 repeats |
| K02F2.3 | tag-203 |  |
| C37A2.4 | cye-1 |  |
| E02D9.1 | E02D9.1 | Eukaryotic protein kinase |
| K02F2.6 | ser-3 | Rhodopsin-like GPCR superfamily |
| T21G5.4 | T21G5.4 | PDZ domain (also known as DHR or GLGF) |
| C48B6.2 | C48B6.2 | Ribosomal protein S4 |
| C48B6.6 | smg-1 |  |
| W01A8.4 | W01A8.4 |  |
| F22D6.5 | prpf-4 | Eukaryotic protein kinase |
| M05B5.5 | hlh-2 | Helix-loop-helix dimerization domain |
| C01H6.2 | C01H6.2 | Ankyrin-repeat |
| R06C7.5 | R06C7.5 | Fumarate lyase |
| R06C7.8 | bub-1 | Eukaryotic protein kinase |
| F21C3.5 | pfd-6 | KE2 family protein |
| C26C6.5 | dcp-66 |  |
| C26C6.1 | pbrm-1 | Zinc finger, C2H2 type |
| C26C6.2 | goa-1 | Guanine nucleotide binding protein (G-protein), alpha subunit |
| D2030.4 | D2030.4 | NDUFB7/B18 subunit of the mitochondrial NADH dehydrogenase (ubiquinone) comple0 |
| F29D11.2 | F29D11.2 | capg-1 organize chromosomal structure and regulate chromosome segregation |
| F26A3.2 | ncbp-2 | RNA-binding region RNP-1 (RNA recognition motif) |
| F20G4.3 | nmy-2 | Myosin head (motor domain) |
| C54G4.8 | cyc-1 | Cytochrome c family heme-binding site |
| K04G2.1 | iftb-1 | Domain found in IF2B/IF5 translation initiation factor 2 beta |
| ZK265.6 | ZK265.6 | Nop16 is a protein involved in ribosome biogenesis. Uncharacterized conserved protein |
| F52B5.6 | rpl-25.2 | Ribosomal L23 protein |
| T01G9.4 | npp-2 |  |
| T01G9.6 | kin-10 | Casein kinase II, regulatory subunit |
| F16D3.4 | F16D3.4 | rapid microtubule elongation 0 |
| T19A6.2 | ngp-1 | GTP1/OBG family Nucleolar GTPase |
| T01H8.5 | gon-2 | Cation channels (non-ligand gated) |
| F43G9.5 | F43G9.5 | Mitochondrial energy transfer proteins (carrier protein) |
| F43G9.1 | F43G9.1 | Isocitrate and isopropylmalate dehydrogenases |
| F43G9.10 | F43G9.10 | Microfibrillar-associated protein 2 0 |
| F43G9.12 | F43G9.12 | Transcriptional regulators binding to the GC-rich sequences0 |
| F39H2.2 | sig-7 | RNA-binding region RNP-1 (RNA recognition motif) |
| C36B1.1 | cle-1 | only vertebrate type XV/XVIII collagen homolog in C. elegans |
| DY3.2 | lmn-1 | nuclear lamin; Intermediate filament tail domain |
| C36B1.5 | prp-4 | U4/U6 small nuclear ribonucleoproteinG-protein beta WD-40 repeats |
| F32H2.1 | gei-11 | Myb DNA binding domain |
| W10D5.2 | nduf-7 | Respiratory-chain NADH dehydrogenase 20 Kd subunit |
| W10D5.3 | gei-17 | MIZ domain (Msx-interacting-zinc finger) |
| ZK858.1 | ZK858.1 | gld-4 Poly(A) polymerases PAP/25A core domain |
| C45G3.3 | gip-2 | Biotin/lipoate A/B protein ligase family |
| F25H5.4 | eft-2 | translation elongation factor 2 |
| F25H5.5 | F25H5.5 | Claspin, an S-phase checkpoint component that interacts with Chk1 and negatively regulates cell cycle progression |
| C45G3.1 | aspm-1 | IQ calmodulin-binding motif |
| F36H2.1 | tat-5 | E1-E2 ATPases aminophospholipid translocase (flippase) |
| F14B4.3 | F14B4.3 | RNA polymerases beta subunit |
| F46A9.4 | skr-2 | SKP1-like ubiquitin-ligase complex that facilitates ubiquitin-mediated protein degradation |
| F46A9.5 | skr-1 | SKP1-like |
| F30A10.10 | F30A10.10 | Ubiquitin carboxyl-terminal hydrolase family 2 |
| K07A1.11 | rba-1 | G-protein beta WD-40 repeats |
| K07A1.12 | lin-53 | G-protein beta WD-40 repeats |
| T05F1.6 | hsr-9 | BRCT domain |
| C03D6.1 | C03D6.1 |  |
| C03D6.8 | rpl-24.2 |  |
| K02A11.1 | gfi-2 | Ankyrin-repeat |
| T23D8.5 | his-67 |  |
| T23D8.6 | his-68 |  |
| T23D8.3 | T23D8.3 |  |
| T23D8.9 | sys-1 | Proline-rich region |
| F10G8.3 | npp-17 | G-protein beta WD-40 repeats |
| C25A1.9 | rsa-1 | EF-hand family |
| Y106G6E.6 | csnk-1 |  |
| Y106G6H.2 | pab-1 | RNA-binding region RNP-1 (RNA recognition motif) |
| F45H11.2 | ned-8 | Ubiquitin domain |
| F25D7.3 | blmp-1 | Zinc finger, C2H2 type |
| Y106G6H.7 | sec-8 |  |
| F25H2.5 | F25H2.5 | Nucleoside diphosphate kinase |
| T22A3.5 | pash-1 | Proline-rich region |
| C43H8.1 | C43H8.1 | Protein of unknown function DUF101 |
| B0511.8 | tag-264 | Cytochrome c family heme-binding site |
| B0511.10 | eif-3.E | Domain in components of the proteasome, COP9-complex and eIF3 (PCI) |
| B0205.7 | kin-3 |  |
| F56G4.4 | F56G4.4 | RNA-binding protein C2H2 Zn-finger domain |
| F15D3.7 | F15D3.7 |  |
| ZK1151.1 | vab-10 | Src homology 3 (SH3) domain |
| R09B3.4 | ubc-12 | Ubiquitin-conjugating enzymes |
| R06C1.3 | wve-1 | Proline-rich region |
| Y53C10A.12 | hsf-1 | Heat shock factor (HSF)-type DNA-binding domain |
| C15C6.3 | C15C6.3 | Proline-rich region |
| C15C6.4 | C15C6.4 | Domain of unknown function UPF0086 |
| W02D9.1 | pri-2 |  |
| T06G6.9 | pfd-3 |  |
| W02A11.1 | W02A11.1 |  |
| W02A11.4 | uba-2 | Repeat in ubiquitin-activating (UBA) protein |
| Y18D10A.13 | pad-1 |  |
| T04D3.2 | sdz-30 | EF-hand family |
| Y40B1A.4 | sptf-3 | Zinc finger, C2H2 type |
| W09C5.1 | W09C5.1 |  |
| W09C5.2 | unc-59 | Cell division GTP binding protein |
| W09C5.6 | rpl-31 | Ribosomal protein L31e |
| W04A4.5 | W04A4.5 |  |
| W04A8.2 | W04A8.2 |  |
| W04A8.7 | taf-1 | Cytochrome c family heme-binding site |
| C37A5.9 | pry-1 |  |
| F49B2.5 | src-2 | Eukaryotic protein kinase |
| Y54E5A.4 | npp-4 |  |
| F39B2.1 | F39B2.1 | Zinc finger, C2H2 type |
| Y54E5B.3 | let-49 |  |
| F33H2.5 | F33H2.5 | DNA-directed DNA polymerase family B |
| F31C3.5 | F31C3.5 |  |
| F23F1.1 | nfyc-1 |  |
| C23H3.5 | C23H3.5 |  |
| D1069.3 | D1069.3 |  |
| C24H12.5 | C24H12.5 |  |
| W07E6.4 | prp-21 | SURP domain |
| W08F4.8 | cdc-37 |  |
| T07D3.7 | alg-2 | PAZ domain |
| M01D1.8 | fbxb-41 | F-box domain |
| F47F6.1 | lin-42 |  |
| F52C6.12 | F52C6.12 |  |
| F52C6.13 | F52C6.13 |  |
| F59H6.7 | cya-2 | Cyclin |
| F07E5.5 | F07E5.5 | Zn-finger CCHC type |
| F42G2.6 | F42G2.6 | Cytochrome b/b6 |
| T22D2.1 | vab-19 | Ankyrin-repeat |
| F09D1.1 | F09D1.1 | Ubiquitin carboxyl-terminal hydrolase family 2 |
| F29A7.6 | F29A7.6 | Vitamin K-dependent carboxylation/gamma-carboxyglutamic (GLA) domain |
| K10G6.1 | lin-31 | Proline-rich region |
| C01F1.1 | C01F1.1 |  |
| C01F1.2 | C01F1.2 | Lipocalin and cytosolic fatty-acid binding protein |
| ZK430.7 | ZK430.7 | G-protein beta WD-40 repeats |
| B0286.4 | ntl-2 |  |
| B0286.5 | fkh-6 | Fork head domain |
| F10G7.4 | scc-1 |  |
| F41C3.4 | F41C3.4 |  |
| C27A2.3 | ify-1 |  |
| C27A2.6 | dsh-2 | Pleckstrin putative G-protein interacting domain |
| F09E5.1 | pkc-3 | Octicosapeptide repeat |
| ZK177.6 | fzy-1 |  |
| C17G10.2 | C17G10.2 | TPR repeat |
| C18A3.3 | C18A3.3 |  |
| F10C1.5 | dmd-5 | DM DNA binding domain |
| ZK1248.11 | ZK1248.11 |  |
| F21H12.5 | fbf-2 | Pumilio-family RNA binding domains (aka PUM-HD, Pumilio homology domain) |
| T24H7.5 | tat-4 | G-protein beta WD-40 repeats |
| K10B2.1 | lin-23 | G-protein beta WD-40 repeats |
| T28D9.2 | rsp-5 |  |
| F18C5.2 | wrn-1 | Pyruvate formate-lyase, glycine radical |
| C56C10.8 | icd-1 | TonB-dependent receptor protein |
| ZK1127.9 | ZK1127.9 |  |
| ZK1127.5 | ZK1127.5 | RNA 3'-terminal phosphate cyclase |
| E04F6.4 | E04F6.4 | Phospholipase D/Transphosphatidylase |
| F45E12.3 | cul-4 | Cullin family |
| R07G3.1 | cdc-42 | Ras family |
| B0495.6 | B0495.6 |  |
| C06A8.2 | C06A8.2 |  |
| C06A8.4 | skr-17 | SKP1-like |
| T05A6.1 | cki-1 | Cyclin-dependent kinase inhibitor |
| T09A5.6 | mdt-10 |  |
| T09A5.9 | T09A5.9 | Leucine-rich repeat |
| T01H3.2 | T01H3.2 |  |
| C08B11.1 | zyg-11 |  |
| C08B11.3 | C08B11.3 | Zinc finger, C2H2 type |
| C08B11.5 | sap-49 |  |
| F10B5.6 | emb-27 | TPR repeat |
| T05C12.7 | cct-1 |  |
| C26D10.1 | ran-3 | Regulator of chromosome condensation (RCC1) |
| K01C8.9 | nst-1 | GTP-binding protein (HSR1-related) |
| C26D10.2 | hel-1 | DEAD/DEAH box helicase |
| C26D10.5 | eff-1 | Phospholipase A2 |
| F22B5.1 | evl-20 |  |
| F22B5.7 | zyg-9 | HEAT repeat |
| T13H5.4 | T13H5.4 | RNA-binding protein C2H2 Zn-finger domain |
| T13H5.5 | T13H5.5 |  |
| F54C9.9 | F54C9.9 |  |
| F54C9.1 | iff-2 | Eukaryotic initiation factor 5A hypusine (eIF-5A) |
| F54C9.5 | rpl-5 | Ribosomal protein L18P/L5E |
| D2085.3 | D2085.3 | Bacterial transferase hexapeptide repeat |
| T14D7.2 | oac-46 |  |
| T21B10.7 | cct-2 | Chaperonins TCP-1 |
| T21B10.3 | T21B10.3 | LIM domain |
| C18E9.4 | C18E9.4 |  |
| F37B12.1 | F37B12.1 |  |
| T23G7.1 | dpl-1 |  |
| C01G6.8 | cam-1 | Kringle domain |
| D2013.7 | eif-3.F | Mov34 family |
| E02H1.1 | E02H1.1 | SAM (and some other nucleotide) binding motif |
| Y53C12B.2 | Y53C12B.2 |  |
| C08H9.2 | C08H9.2 | KH domain |
| C08H9.3 | C08H9.3 |  |
| R53.3 | egl-43 | Zinc finger, C2H2 type |
| R53.4 | R53.4 |  |
| R53.6 | R53.6 |  |
| R53.7 | R53.7 | CBS domain |
| F27E5.2 | pax-3 | Homeobox domain |
| ZK970.2 | clpp-1 | Clp protease |
| ZK970.3 | mdt-22 | Clp protease |
| C07E3.1 | stip-1 | D111/G-patch domain |
| R166.4 | pro-1 | Regulator of chromosome condensation (RCC1) |
| F59B10.1 | pqn-47 |  |
| R06F6.1 | cdl-1 |  |
| T19E10.1 | ect-2 | Dbl domain (dbl/cdc24 rhoGRF family) |
| M106.5 | cap-2 | F-actin capping protein beta subunit |
| F59E10.1 | orc-2 |  |
| C09H10.2 | rpl-41 | Forkhead-associated (FHA) domain |
| C09H10.7 | C09H10.7 |  |
| C09H10.8 | glb-4 |  |
| W02B12.2 | rsp-2 | RNA-binding region RNP-1 (RNA recognition motif) |
| F54D5.8 | dnj-13 | DnaJ N-terminal domain |
| C47D12.1 | trr-1 | Phosphatidylinositol 3- and 4-kinase |
| ZK930.3 | ZK930.3 |  |
| W03C9.4 | lin-29 | Zinc finger, C2H2 type |
| Y17G7B.5 | mcm-2 | MCM family |
| C50E10.4 | sop-2 |  |
| Y48E1B.5 | Y48E1B.5 |  |
| ZK131.8 | his-14 |  |
| ZK131.9 | his-15 |  |
| ZK131.1 | his-26 |  |
| ZK131.4 | his-10 |  |
| ZK131.5 | his-11 |  |
| F08G2.1 | his-44 |  |
| Y51H1A.6 | mcd-1 | Zinc finger, C2H2 type |
| W01G7.3 | rpb-11 | RNA polymerases L/13 to 16 Kd subunits |
| K09E4.1 | K09E4.1 |  |
| W03H9.4 | cacn-1 |  |
| Y48B6A.1 | Y48B6A.1 | G-protein beta WD-40 repeats |
| F26H11.1 | kbp-3 |  |
| Y54E2A.1 | Y54E2A.1 | Rhodopsin-like GPCR superfamily |
| W05G11.2 | W05G11.2 |  |
| W07B3.2 | gei-4 |  |
| F10C5.1 | mat-3 |  |
| C09F5.1 | C09F5.1 |  |
| W06E11.2 | tag-267 |  |
| F58B6.3 | par-2 |  |
| F56F11.4 | F56F11.4 | AAA-protein (ATPases associated with various cellular activities) |
| F59A2.1 | npp-9 | RanBP1 domain |
| F59A2.4 | F59A2.4 |  |
| C32A3.1 | sel-8 |  |
| C54C6.1 | rpl-37 | Ribosomal protein L37e |
| C54C6.2 | ben-1 | Tubulin family |
| T02C12.2 | T02C12.2 |  |
| C03C10.3 | rnr-2 | Ribonucleotide reductase |
| F43C1.2 | mpk-1 | Eukaryotic protein kinase |
| Y44F5A.1 | Y44F5A.1 | G-protein beta WD-40 repeats |
| T08A11.2 | T08A11.2 |  |
| B0285.1 | B0285.1 | Eukaryotic protein kinase |
| H38K22.1 | evl-14 |  |
| H38K22.2 | dcn-1 |  |
| R07E5.3 | R07E5.3 |  |
| R07E5.10 | pdcd-2 | MYND zinc finger (ZnF) domain |
| R07E5.14 | rnp-4 | RNA-binding region RNP-1 (RNA recognition motif) |
| F56F3.5 | rps-1 | Ribosomal protein S3Ae |
| F56F3.6 | ins-17 | Insulin/IGF/Relaxin family |
| C07G2.3 | cct-5 | Chaperonins TCP-1 |
| F35G12.2 | F35G12.2 | Isocitrate and isopropylmalate dehydrogenases |
| F35G12.8 | smc-4 | ABC transporters family |
| B0393.1 | rps-0 | Ribosomal protein S2 |
| F26F4.11 | rpb-8 |  |
| C26E6.8 | ula-1 | UBA/THIF-type NAD/FAD binding fold |
| R144.7 | larp-1 |  |
| F54D8.1 | dpy-17 |  |
| T10F2.4 | T10F2.4 | G-protein beta WD-40 repeats |
| C34E10.2 | gop-2 | ABC transporters family |
| R02F2.7 | R02F2.7 |  |
| F56D2.6 | F56D2.6 | DEAD/DEAH box helicase |
| F54E7.3 | par-3 |  |
| B0336.6 | abi-1 | Src homology 3 (SH3) domain |
| B0336.2 | arf-1.2 |  |
| F01F1.7 | ddx-23 |  |
| F01F1.8 | cct-6 | Chaperonins TCP-1 |
| F25B5.2 | F25B5.2 |  |
| C23G10.8 | C23G10.8 |  |
| T12A2.7 | T12A2.7 |  |
| F47D12.4 | hmg-1.2 |  |
| C05D11.3 | tag-170 | Thioredoxin family |
| C16A3.6 | C16A3.6 |  |
| T26A5.9 | dlc-1 | Dynein light chain type 1 |
| F20H11.2 | nsh-1 |  |
| F20H11.6 | F20H11.6 |  |
| F37A4.6 | F37A4.6 |  |
| F37A4.8 | isw-1 | SNF2 related domain |
| K07E12.1 | dig-1 | Aspartic acid and asparagine hydroxylation site |
| R13F6.1 | kbp-1 |  |
| K04C2.2 | K04C2.2 |  |
| F57B9.5 | byn-1 |  |
| F11H8.4 | cyk-1 | Proline-rich region |
| K04G7.10 | rnp-7 | RNA-binding region RNP-1 (RNA recognition motif) |
| R151.9 | pfd-5 |  |
| T20B12.1 | T20B12.1 | TPR repeat |
| T20B12.8 | hmg-4 | HMG1/2 (high mobility group) box |
| C07H6.5 | cgh-1 |  |
| R13A5.12 | lpd-7 |  |
| ZK686.1 | ZK686.1 |  |
| C03B8.4 | lin-13 | Zinc finger, C2H2 type |
| ZK686.3 | ZK686.3 |  |
| C29E4.7 | gsto-1 | Glutathione S-transferase |
| C29E4.8 | let-754 | Adenylate kinase |
| C29E4.2 | kle-2 |  |
| F54H12.1 | aco-2 | RNA-binding region RNP-1 (RNA recognition motif) |
| K12H4.3 | K12H4.3 |  |
| K12H4.5 | K12H4.5 |  |
| K06H7.6 | apc-2 |  |
| K12H4.8 | dcr-1 |  |
| C14B9.4 | plk-1 | POLO box duplicated region |
| C50C3.6 | prp-8 |  |
| C02F5.1 | knl-1 |  |
| F09G8.3 | F09G8.3 |  |
| F10E9.5 | F10E9.5 |  |
| R05D3.11 | met-2 |  |
| R05D3.4 | rfp-1 | Thioredoxin family |
| ZK1236.3 | sor-1 | Proline-rich region |
| C30C11.1 | C30C11.1 |  |
| C30C11.4 | C30C11.4 | Heat shock protein hsp70 |
| PAR2.4 | mig-22 |  |
| F54F2.2 | zfp-1 |  |
| ZK637.7 | lin-9 |  |
| ZK637.8 | unc-32 | V-type ATPase 116kDa subunit family |
| R08D7.1 | R08D7.1 |  |
| R08D7.2 | R08D7.2 |  |
| R08D7.3 | eif-3.D |  |
| R107.8 | lin-12 | Aspartic acid and asparagine hydroxylation site |
| ZK507.6 | ZK507.6 | Cyclin |
| F54G8.3 | ina-1 | Integrins alpha chain |
| T23G5.1 | rnr-1 | Ribonucleotide reductase large subunit |
| F54C8.3 | emb-30 |  |
| T26G10.1 | T26G10.1 | ATP-dependent helicase, DEAD-box |
| F54C8.2 | cpar-1 | Histone H3 |
| B0464.5 | spk-1 | Eukaryotic protein kinase |
| B0464.7 | baf-1 |  |
| F58A4.8 | tbg-1 | Tubulin family |
| F58A4.3 | hcp-3 | Histone H3 |
| F58A4.4 | pri-1 | DNA primase small subunit |
| C15H7.4 | C15H7.4 |  |
| C07A9.2 | C07A9.2 | G10 protein |
| T05G5.2 | hlh-4 | Proline-rich region |
| T05G5.3 | cdk-1 | Eukaryotic protein kinase |
| ZK632.1 | mcm-6 | MCM family |
| ZK632.2 | ZK632.2 | Forkhead-associated (FHA) domain |
| F40F12.7 | F40F12.7 | TAZ finger |
| K03H1.10 | K03H1.10 | TAZ finger |
| T16G12.5 | ekl-6 |  |
| ZK1128.3 | ZK1128.3 |  |
| ZK1128.5 | tag-246 | BAF60b domain of the SWIB complex |
| K10G9.2 | K10G9.2 |  |
| D2045.9 | D2045.9 | LPS glycosyltransferase |
| Y39A1A.13 | Y39A1A.13 |  |
| Y39A1A.12 | Y39A1A.12 | AAA-protein (ATPases associated with various cellular activities) |
| Y48A6C.2 | Y48A6C.2 |  |
| Y48A6B.11 | rsa-2 |  |
| Y48A6C.5 | pha-1 |  |
| T28D6.5 | T28D6.5 | Myb DNA binding domain |
| T28D6.6 | T28D6.6 | GTP1/OBG family |
| Y47D3B.7 | sbp-1 | Helix-loop-helix dimerization domain |
| Y66A7A.5 | Y66A7A.5 | Homeobox domain |
| Y41C4A.10 | elb-1 |  |
| C18D11.4 | rsp-8 | RNA-binding region RNP-1 (RNA recognition motif) |
| Y56A3A.4 | taf-12 | Histone-fold/TFIID-TAF/NF-Y domain |
| Y56A3A.6 | Y56A3A.6 | Proline-rich region |
| Y75B8A.7 | Y75B8A.7 |  |
| Y56A3A.20 | ccf-1 |  |
| Y49E10.6 | his-72 | Cell division GTP binding protein |
| Y49E10.20 | Y49E10.20 | CD36 family |
| Y49E10.21 | Y49E10.21 |  |
| Y37D8A.9 | mrg-1 |  |
| Y37D8A.11 | Y37D8A.11 |  |
| Y39E4B.1 | abce-1 | 4Fe-4S ferredoxin, iron-sulfur binding domain |
| Y43F4B.6 | klp-19 | Kinesin motor domain |
| F53A2.4 | nud-1 |  |
| F45G2.8 | F45G2.8 |  |
| T05D4.4 | osm-7 |  |
| T25C8.2 | act-5 | Actin |
| W06F12.1 | lit-1 | Eukaryotic protein kinase |
| K08E3.6 | cyk-4 | RhoGAP domain |
| F29C4.2 | F29C4.2 |  |
| R02D3.5 | R02D3.5 | Protein prenyltransferases alpha subunit repeat |
| F56A11.1 | gex-2 |  |
| F56B3.4 | F56B3.4 | SKP1-like |
| C50A2.2 | C50A2.2 |  |
| F53H1.1 | F53H1.1 |  |
| F55A8.1 | egl-18 | GATA-type zinc finger domain |
| C37F5.1 | lin-1 |  |
| F42A6.7 | hrp-1 |  |
| R08C7.3 | htz-1 | Histone-fold/TFIID-TAF/NF-Y domain |
| R08C7.4 | R08C7.4 |  |
| F29B9.6 | ubc-9 |  |
| E04A4.5 | E04A4.5 |  |
| T19E7.3 | bec-1 |  |
| B0547.1 | csn-5 | Mov34 family |
| C06E7.1 | C06E7.1 |  |
| C06E7.3 | C06E7.3 | S-adenosylmethionine synthetase |
| K08B4.1 | lag-1 | IPT/TIG domain |
| T13A10.11 | tag-32 | S-adenosylmethionine synthetase |
| C43G2.2 | bicd-1 |  |
| T22D1.10 | ruvb-2 |  |
| C06G3.10 | cogc-2 |  |
| C42D4.8 | rpc-1 | RNA polymerase, alpha subunit |
| C06E4.6 | C06E4.6 | Short-chain dehydrogenase/reductase (SDR) superfamily |
| C48A7.1 | egl-19 | Cation channels (non-ligand gated) |
| C48A7.2 | C48A7.2 | Clathrin adaptor complex, small chain |
| F55G1.10 | his-61 |  |
| F55G1.3 | his-62 | Histone-fold/TFIID-TAF/NF-Y domain |
| F55G1.11 | his-60 |  |
| C46A5.3 | col-14 | Collagen triple helix repeat |
| C46A5.4 | C46A5.4 | Aldo/keto reductase family |
| C46A5.5 | C46A5.5 |  |
| C33H5.7 | C33H5.7 | G-protein beta WD-40 repeats |
| C33H5.9 | sec-10 |  |
| C33H5.12 | rsp-6 | RNA-binding region RNP-1 (RNA recognition motif) |
| F57H12.1 | arf-3 |  |
| F42G8.3 | pmk-2 | Eukaryotic protein kinase |
| F42G8.6 | F42G8.6 | NAD binding site |
| F35H10.4 | vha-5 | V-type ATPase 116kDa subunit family |
| F17E9.12 | his-31 |  |
| F17E9.9 | his-34 |  |
| D2096.8 | D2096.8 | Nucleosome assembly protein (NAP) |
| T26A8.4 | T26A8.4 | Zinc finger C-x8-C-x5-C-x3-H type |
| F08B4.5 | F08B4.5 |  |
| ZC168.3 | ZC168.3 |  |
| K01H12.2 | ant-1.3 | Mitochondrial energy transfer proteins (carrier protein) |
| ZK1251.9 | ZK1251.9 |  |
| T13F2.7 | sna-2 |  |
| W08D2.7 | mtr-4 | DEAD/DEAH box helicase |
| K07F5.14 | K07F5.14 |  |
| F25H8.2 | F25H8.2 |  |
| F25H8.3 | gon-1 | Neutral zinc metallopeptidases, zinc-binding region |
| W01B6.9 | ndc-80 |  |
| C04H5.6 | mog-4 | DEAD/DEAH box helicase |
| R09E10.7 | pqn-55 |  |
| R10H10.2 | spe-26 | Kelch repeat |
| F23B2.13 | rpb-12 | Cytochrome c family heme-binding site |
| T11G6.8 | T11G6.8 | RNA-binding region RNP-1 (RNA recognition motif) |
| F36H1.2 | tag-144 | Aminoacyl-transfer RNA synthetases class-I |
| M7.1 | let-70 | Ubiquitin-conjugating enzymes |
| F01G4.1 | psa-4 | SNF2 related domain |
| F01G4.2 | ard-1 | Eukaryotic thiol (cysteine) proteases active sites |
| F01G4.6 | F01G4.6 | Regulator of chromosome condensation (RCC1) |
| C08F8.1 | pfd-1 |  |
| C08F8.8 | nhr-67 | Vitamin D receptor |
| B0035.8 | his-48 |  |
| F54E12.3 | his-56 |  |
| B0035.9 | his-46 |  |
| F54E12.4 | his-58 |  |
| B0035.11 | B0035.11 |  |
| H02I12.6 | his-66 | Histone-fold/TFIID-TAF/NF-Y domain |
| F22B3.1 | his-64 |  |
| C10C6.2 | npr-3 | Rhodopsin-like GPCR superfamily |
| M04B2.1 | mep-1 | Zinc finger, C2H2 type |
| F12F6.7 | F12F6.7 |  |
| ZK809.4 | ent-1 | Delayed-early response protein/equilibrative nucleoside transporter |
| ZK792.6 | let-60 | Ras family |
| K08E4.1 | spt-5 |  |
| F11A10.2 | F11A10.2 | RNA-binding region RNP-1 (RNA recognition motif) |
| B0001.7 | B0001.7 |  |
| F28D1.7 | rps-23 | Ribosomal protein S12 |
| F28D1.10 | gex-3 |  |
| C25G4.6 | C25G4.6 | PDZ domain (also known as DHR or GLGF) |
| T23F6.4 | rbd-1 | RNA-binding region RNP-1 (RNA recognition motif) |
| C39E9.13 | rfc-3 | Replication factor C conserved domain |
| C39E9.14 | dli-1 | Ras family |
| JC8.6 | lin-54 |  |
| K09B11.3 | K09B11.3 |  |
| Y45F10D.9 | sas-6 |  |
| B0513.1 | lin-66 |  |
| Y116A8C.35 | uaf-2 | RNA-binding region RNP-1 (RNA recognition motif) |
| Y116A8C.32 | sfa-1 | Proline-rich region |
| W03F9.10 | W03F9.10 |  |
| T10B5.5 | cct-7 |  |
| T10B5.6 | knl-3 |  |
| F02C9.3 | tat-6 | E1-E2 ATPases |
| T22F3.4 | rpl-11.1 | Ribosomal protein L5 |
| T27C4.4 | lin-40 |  |
| F32D1.10 | mcm-7 | MCM family |
| K03B4.1 | K03B4.1 |  |
| R02F11.4 | R02F11.4 | Leucine-rich repeat |
| C37H5.6 | C37H5.6 | Adenylosuccinate synthetase |
| B0238.11 | B0238.11 |  |
| CD4.4 | vps-37 |  |
| F25B4.9 | clec-1 | C-type lectin domain |
| F26F12.7 | let-418 | SNF2 related domain |
| F29G9.4 | fos-1 | bZIP (Basic-leucine zipper) transcription factor family |
| F44C4.4 | gon-14 |  |
| ZC404.7 | ZC404.7 |  |
| F09G2.4 | cpsf-2 |  |
| F19F10.9 | F19F10.9 |  |
| C37C3.6 | ppn-1 |  |
| F26D11.11 | let-413 | PDZ domain (also known as DHR or GLGF) |
| W02D7.7 | sel-9 | emp24/gp25L/p24 family |
| H24G06.1 | H24G06.1 | G-protein beta WD-40 repeats |
| F11A3.2 | F11A3.2 | Initiation factor 2B |
| C50F4.11 | mdf-1 | NUDIX hydrolase domain |
| C50F4.5 | his-41 | Histone-fold/TFIID-TAF/NF-Y domain |
| C50F4.7 | his-37 |  |
| F25B3.6 | F25B3.6 |  |
| ZK287.5 | rbx-1 | RING finger |
| C27H6.2 | ruvb-1 |  |
| ZK856.10 | ZK856.10 |  |
| K07C5.4 | K07C5.4 | Putative snoRNA binding domain |
| Y32F6A.3 | pap-1 | PAP/25A core domain |
| F53B7.3 | F53B7.3 |  |
| T04C12.4 | act-3 |  |
| T04C12.5 | act-2 | Actin |
| T04C12.6 | act-1 |  |
| C06H2.1 | atp-5 |  |
| C55A6.9 | C55A6.9 |  |
| T27F2.1 | skp-1 |  |
| F55A11.2 | syn-3 | Syntaxin / epimorphin family |
| F55C5.4 | F55C5.4 |  |
| F55C5.8 | F55C5.8 |  |
| F18E2.2 | abcf-1 | ABC transporters family |
| F18E2.3 | scc-3 |  |
| F53F4.11 | F53F4.11 |  |
| F58E10.2 | end-1 | GATA-type zinc finger domain |
| H39E23.1 | par-1 | Ubiquitin-associated domain |
| C53A5.3 | hda-1 | Histone deacetylase family |
| F43D2.1 | F43D2.1 |  |
| T01C3.1 | T01C3.1 | G-protein beta WD-40 repeats |
| C25D7.6 | mcm-3 | MCM family |
| T06E6.1 | T06E6.1 | EF-hand family |
| T06E6.2 | cyb-3 | Cyclin |
| T26H5.5 | srw-33 | Rhodopsin-like GPCR superfamily |
| C06B8.8 | rpl-38 | Ribosomal L38e protein family |
| F28F8.5 | F28F8.5 |  |
| T10C6.11 | his-4 | Histone-fold/TFIID-TAF/NF-Y domain |
| T10C6.14 | his-1 |  |
| ZK262.8 | ZK262.8 | Myosin head (motor domain) |
| Y51A2D.15 | Y51A2D.15 |  |
| Y113G7B.23 | psa-1 | Proline-rich region |
| F38A6.1 | pha-4 | Fork head domain |
| F07D10.1 | rpl-11.2 | Ribosomal protein L5 |
| T14F9.4 | peb-1 | Crystallin |
| C54D1.6 | bar-1 |  |
| K03A1.6 | his-38 |  |
| K08A8.2 | sox-2 | HMG1/2 (high mobility group) box |
| C47C12.3 | ref-2 | Zinc finger, C2H2 type |
| C14F5.5 | sem-5 | Src homology 2 (SH2) domain |
| F08F1.9 | F08F1.9 | Cyclin |
| D2021.1 | utx-1 | TPR repeat |
| F15G9.4 | him-4 | Aspartic acid and asparagine hydroxylation site |
| F47A4.2 | dpy-22 | RNA-binding region RNP-1 (RNA recognition motif) |
| F44A6.2 | sex-1 | Ligand-binding domain of nuclear hormone receptor |
| ZC504.4 | mig-15 | Eukaryotic protein kinase |
| F13E6.4 | F13E6.4 | WW / rsp5 / WWP domain |
| F58A3.1 | ldb-1 | Proline-rich region |
| F58A3.2 | egl-15 | Eukaryotic protein kinase |
| T25C12.1 | lin-14 |  |
| C35C5.1 | sdc-2 |  |
| F19H6.1 | nekl-3 | Eukaryotic protein kinase |
| F18H3.5 | cdk-4 | Eukaryotic protein kinase |
| F48F7.1 | alg-1 | PAZ domain |
| C11H1.3 | C11H1.3 | RING finger |
| K09A9.3 | ent-2 | Delayed-early response protein/equilibrative nucleoside transporter |
| H30A04.1 | eat-20 | EGF-like domain |
| F11C7.5 | osm-11 |  |
| F31A3.4 | hlh-29 | Helix-loop-helix dimerization domain |
| Y65B4BR.5 | Y65B4BR.5 |  |
| C09G12.8 | ced-10 | ced-10 encodes a GTPase orthologous to human RAC1 (OMIM:602048) |
| F54E12.3 | his-56 | his-56 encodes an H4 histone. |
| F15D3.7 | F15D3.7 |  |
| Y56A3A.20 | ccf-1 | a homolog of subunit 7 of CCR4-NOT transcription complex from S. cerevisiae. |
| B0205.7 | kin-3 | kin-3 encodes an ortholog of the catalytic subunit of casein kinase II alpha |
| Y47G6A.8 | crn-1 |  |
| Y49F6B.1 | cyh-1 |  |
| Y50E8A.4 | unc-61 |  |
| M03F4.2 | act-4 | actin |
| F56F11.4 | F56F11.4 |  |
| F45F2.3 | his-5 | his-5 encodes an H4 histone. |
| F55D10.2 | rpl-25.1 | rpl-25.1 encodes a large ribosomal subunit L23a protein. |
| Y71H2B.6 | mdt-19 |  |
| C26B9.3 | C26B9.3 |  |
| Y57A10A.19 | rsr-2 |  |
| T28F12.2 | unc-62 |  |
| Y71F9AM.5 | nxt-1 |  |
| Y105E8B.1 | lev-11 | lev-11 tropomyosin, an actin-binding contractile structural protein orthologous to human TROPOMYOSIN 1 |
| C54H2.5 | sft-4 |  |
| B0207.4 | air-2 | air-2 encodes an aurora/Ip11-related serine/threonine protein kinase |
| ZK154.3 | mec-7 | mec-7 encodes a beta-tubulin |
| Y111B2A.15 | tpst-1 |  |
| T22B7.1 | egl-13 | egl-13 encodes a transcription factor of the HMG (high mobility group) box family |
| Y92C3B.2 | uaf-1 |  |
| F13D11.2 | hbl-1 | hbl-1 encodes a protein that contains nine putative zinc finger domains of the C2H2 type |
| Y48G8AL.6 | smg-2 |  |
| F35C8.7 | chtl-1 |  |
| Y92C3B.1 | kbp-4 |  |
| F45G2.8 | F45G2.8 |  |
| Y95D11A.1 | Y95D11A.1 |  |
| Y38F2AL.4 | vha-3 |  |
| Y65B4BR.8 | Y65B4BR.8 |  |
| Y110A7A.14 | pas-3 |  |
| T11G6.8 | T11G6.8 |  |
| Y56A3A.1 | ntl-3 | ntl-3 is the ortholog of NOT3/NOT5 |
| F40F12.7 | F40F12.7 |  |
| T05G5.2 | hlh-4 |  |
| Y34D9A.4 | spd-1 |  |
| W09C5.6 | rpl-31 | rpl-31 encodes a large ribosomal subunit L31 protein. |
| Y54E10BL.1 | Y54E10BL.1 | |
| Y54E10BR.4 | Y54E10BR.4 | |
| Y51H7C.6 | cogc-4 |  |
| Y55F3AM.3 | Y55F3AM.3 |  |
| Y47D3A.6 | tra-1 | The tra-1 gene encodes a homolog of human GLI3 |
| W03F9.10 | W03F9.10 |  |
| C38C3.5 | unc-60 | unc-60 encodes several isoforms of an actin depolymerizing factor (ADF)/cofilin homologue |
| Y110A7A.8 | Y110A7A.8 |  |
| F58D5.1 | hrp-2 |  |
| Y50D7A.11 | Y50D7A.11 |  |
| Y71H2B.3 | ppfr-4 |  |
| Y47D3A.26 | smc-3 |  |
| C01G8.5 | erm-1 | ERM family of cytoskeletal linkers with approximately equal similarity to ezrin, radixin and moesin |
| C01G8.9 | let-526 |  |
| Y87G2A.4 | aex-6 |  |
| H27M09.2 | rpb-5 |  |
| Y47G6A.9 | Y47G6A.9 |  |
| Y54E10BL.2 | col-48 |  |
| Y110A7A.13 | chp-1 | chp-1 encodes a protein containing two CHORD domains |
| Y47G6A.20 | rnp-6 | rnp-6 an ortholog of HALF-PINT in D. melanogaster and FBP Interacting Repressor (FIR)/PUF60 in mammals |
| Y38C1AA.4 | tcl-2 |  |
| Y54E10BL.6 | mek-2 |  |
| Y65B4A.6 | Y65B4A.6 |  |
| Y87G2A.1 | Y87G2A.1 |  |
| Y23H5B.5 | Y23H5B.5 |  |
| H27M09.1 | H27M09.1 |  |
| Y54E10A.2 | cogc-1 |  |
| Y110A7A.17 | mat-1 |  |
| Y48G1C.7 | Y48G1C.7 |  |
| Y54E10A.9 | vbh-1 |  |
| Y23H5B.6 | Y23H5B.6 |  |
| Y47G6A.12 | sep-1 | The sep-1 gene encodes a homolog of separase |
| Y48G1C.8 | Y48G1C.8 |  |
| Y110A7A.1 | hcp-6 |  |
| Y38E10A.3 | Y38E10A.3 |  |
| Y57A10A.27 | Y57A10A.27 |  |
| Y17G7B.2 | ash-2 |  |
| Y51H1A.6 | mcd-1 |  |
| C15F1.3 | tra-2 | The tra-2 gene encodes a transmembrane receptor that functions in the sex determination pathway |
| Y77E11A.9 | clec-171 |  |
| Y54G2A.17 | Y54G2A.17 |  |
| Y55F3AR.3 | cct-8 |  |
| Y73F8A.24 | Y73F8A.24 |  |
| Y55F3AM.15 | csn-4 | csn-4 encodes a protein with similarity to human COP9 proteasome subunit 4. |
| F19B6.1 | F19B6.1 |  |
| Y77E11A.7 | Y77E11A.7 |  |
| F45E4.7 | F45E4.7 |  |
| F45F2.12 | his-8 | his-8 encodes an H2B histone; his-8 is contained within the histone gene cluster HIS2. |
| Y111B2A.22 | ssl-1 | The protein product of this gene is predicted to contain a glutamine/asparagine (Q/N)-rich ('prion') domain |
| Y59A8A.1 | csn-1 | csn-1 encodes an ortholog of subunit 1 of the COP9 signalosome complex (CSN1) |
| Y105E8A.17 | ekl-4 |  |
| K07C5.8 | cash-1 |  |
| Y51A2D.7 | Y51A2D.7 |  |
| Y113G7B.23 | psa-1 | The psa-1 (phasmid socket absent) gene encodes a ortholog of SWI3, a component of the SWI/SNF complex |
| M03F4.6 | M03F4.6 |  |
| F49H12.1 | lsy-2 |  |
| F09F9.4 | F09F9.4 |  |
| C42D8.5 | acn-1 |  |
| Y69A2AR.30 | mdf-2 |  |
| Y54E10A.15 | cdt-1 |  |
| H20J04.4 | H20J04.4 |  |
| F41E6.4 | smk-1 |  |
| C03A7.2 | C03A7.2 |  |
| Y53H1C.2 | ego-2 |  |
| K04G7.11 | K04G7.11 |  |
| Y48A6C.2 | Y48A6C.2 |  |
| Y48G1A.5 | xpo-2 | imb-5 encodes an importin-beta-like protein |
| H32C10.1 | H32C10.1 |  |
| Y105C5B.12 | Y105C5B.12 |  |
| Y111B2A.18 | rsp-3 |  |
| ZK858.1 | ZK858.1 | PAP/25A core domain |
| B0035.9 | his-46 |  |
| C54D1.6 | bar-1 |  |
