# Supplementary material for: An In Vivo EGF Receptor Localization Screen in C. elegans Identifies the Ezrin Homolog ERM-1 as a Temporal Regulator of Signaling
Source: PLoS Genet. 2014 May 1;10(5):e1004341. doi: 10.1371/journal.pgen.1004341 (PMC4006739; doi:10.1371/journal.pgen.1004341)
Supplement: Table S2 — Sequences of primers used. (DOCX) [file pgen.1004341.s006.docx]

| PRIMER NAME | PRIMER SEQUENCE |
| --- | --- |
| OJE-56 (attB4 adaptor sites) | ATAGAAAAGTTGCATCTGCACTTGGGATTCAC |
| OJE-51(attB1r adaptor sites) | TGTACAAACTTGAAGTACTTCTAGACACAC |
| OJE-52(attB1 adaptor sites) | AAA AAGCAGGCTTTATAGAATGGGCAC |
| OJE-53(attB2 adaptor sites) | AGAAAGCTGGGTGAATGCATACCCATTGG |
| OJE-54(attB2r adaptor sites) | TGTACAAAGTGGCATTCACTTGAATGGATG |
| OJE-55(attB3 adaptor sites) | ATAATAAAGTTGCCAGCGACGATATCCATTAGTGC |
| OCH-43(attB4) | ggggacaactttgtATAGAAAAGTTG |
| OCH-42(attB1r) | ggggactgctttttTGTACAAACTTG |
| OCH-38(attB1) | ggggacaagtttgtacaAAAAAGCAGGCT |
| OCH-39(attP2) | ggggaccactttgtacaAGAAAGCTGGGT |
| OCH-40(attB2r) | ggggacagctttctTGTACAAAGTGG |
| OCH-41(attB3) | ggggacaactttgtATAATAAAGTTG |
| OAB-3(attB4) | ATAGAAAAGTTGCCTACCAGCTGGACTGGC |
| OAB-4(attB1r) | TGTACAAACTTGGCAGAAGTCGTGCTGAAC |
| OAB-5(attB1) | AAAAAGCAGGCTGTTCAGCACGACTTCTGCC |
| OAB-7(attB2) | AGAAAGCTGGGTCCGCATCTGGTCAATG |
| OAB-6(attB2R) | TGTACAAAGTGGCATTGACCAGATGCGG |
| OAB-9(attB3) | ATAATAAAGTTGGTTCCAACAATTAATCC |
| OEH-33 | ATGGAGGCTCTTCCAATAATG |
| OEH-34 | AGTCGACCTGCAGGCATGCAAGCTTTGATGACGTGGCGTCATG |
| OEH-35 | GAGCCGATTGATTGAGGTTTTC |
| OEH-36 | GTGAGATGGCTGAAAATTAGAC |
| OEH-37 | CATTATTGGAAGAGCCTCCATTTTTGAGCTGAAATTTTGG |
| FIRE D | AAGGGCCCGTACGGCCGACTAGTAGG |
| FIRE D* | GGAAACAGTTATGTTTGGTATATTGGG |
| OEH-41 | GGAAATTGCAGATTTATAGTAG |
| OEH-47 | AGTCGACCTGCAGGCATGCAAGCTTTTCCAAGAGCTGAAAAAATAG |
| OEH-44 | GCATGGATGAACTATACAAAATGAACCAGTTCCGGGCTC |
| OEH-45 | CTATGTGCATCATTTTGATGAC |
| OJE-05 | AGCTTGCATGCCTGCAGGTCGACT |
| OEH-48 | TTTGTATAGTTCATCCATGCCATG |
| OEH-42 | GAAAAAGTATAAGACCAGGG |
| OEH-46 | CATCTTACAAGCACTTTCTGG |
| OEH-38 | CTGTTTTCAAAAAAATATTAGG |
| OEH-40 | AGTCGACCTGCAGGCATGCAAGCTATTCTTATTCTGCTCCAAAAGT |
| OEH-39 | CAACAATTTTCAACTTCAAG |
