# Supplementary material for: An In Vivo EGF Receptor Localization Screen in C. elegans Identifies the Ezrin Homolog ERM-1 as a Temporal Regulator of Signaling
Source: PLoS Genet. 2014 May 1;10(5):e1004341. doi: 10.1371/journal.pgen.1004341 (PMC4006739; doi:10.1371/journal.pgen.1004341)
Supplement: Text S1 — Details on the construction of the translational reporter constructs used in this study. A schematic drawing of the LET-23::GFP and ERM-1::mCherry constructs is shown in Figure S4A, and the sequences of primers used are listed in Table S2. (DOC) [file pgen.1004341.s007.doc]

Supplementary Methods

Translational reporter constructs

Primers used to generate the constructs are listed in Supplementary Table S2.

let-23::gfp

Three fragments were amplified with primers containing attB sites from genomic let-23 or from a let-23 fragment fused to the gfp sequence (Supplementary Figure S4A). Individual fragments were cloned into Gateway vectors pDONRP4-P1r, pDONR221 and pDONRP2r-P3 (Invitrogen) and recombined into MoSCI destination vector pCFJ150 [1] containing the unc-119 gene. The resulting expression clone vector pJE05 was then integrated into unc-119(-) worms by particle bombardment resulting in the worm line zhIs038[let-23::gfp;unc-119(+)].

erm-1::mCherry

Three fragments were amplified with primers containing attB sites from genomic erm-1 or from a genomic erm-1 fragment fused in frame at the C-terminus to the mCherry sequence (Supplementary Figure S4B). Individual fragments were cloned into Gateway vectors pDONRP4-P1r, pDONR221 and pDONRP2r-P3 (Invitrogen) and recombined into MoSCI destination vector pCFJ150 [1] containing the unc-119 gene. The corresponding vector was injected in the gonads of zhIs038[let-23::gfp;unc-119(+)] and the gonads of erm-1(tm677)/hT2 animals along with the myo-2::mCherry co-injection marker. The resulting extra-chromosomal array rescued the lethality of erm-1(tm677) animals (data not shown).

ego-2::gfp

A 1,4 kb genomic fragment upstream of ego-2 was amplified with primers OEH-35 and OEH-37. A 4,4 kb fragment containing *ego-*2 cDNA was amplified with primers OEH-33 and OEH-34. A 1,7 kb fragment from vector pPD95_75 containing gfp::unc-54 3’UTR was amplified with primers OJE-05 and FIRE D. The three fragments were assembled by fusion PCR [2] using nested primers OEH-36 and FIRE D*. The resulting 7,2 kb fragment was injected into N2 wild type animals along with the myo-2::mCherry co-injection marker.

gfp::sft-4

A 2,6 genomic fragment upstream of sft-4 was amplified with primers OEH-41 and OEH-47. A 2 kb fragment containing the ORF, introns and annotated 3’UTR of sft-4 was amplified with primers OEH-44 and OEH-45. A 1 kb fragment from vector pPD95.75 containing gfp was amplified with primers OJE-05 and OEH-48. The three fragments were assembled by fusion PCR [2] using nested primers OEH-42 and OEH-46 in the following order: 2,7 kb promoter fragment/1 kb gfp/2 kb sft-4 genomic. The resulting 5,7 kb fragment was injected into N2 wild type animals along with the myo-2::mCherry co-injection marker.

C11H1.3::gfp

A 5.6 kb genomic fragment containing 2.2 kb upstream of the ATG and 3.4 kb downstream from the ATG before the stop codon of C11H1.3 was amplified with primers OEH-38 and OEH-40. A 1,7 kb fragment from vector pPD95_75 containing gfp::unc-54 3’UTR was amplified with primers OJE-05 and FIRE D. The three fragments were assembled by fusion PCR [2] using nested primers OEH-39 and FIRE D*. The resulting 7,3 kb fragment was injected into N2 wild type animals along with the myo-2::mCherry co-injection marker.

**Additional references to the supplementary methods**

1. Frøkjær-Jensen C, Davis MW, Hopkins CE, Newman BJ, Thummel JM, et al. (2008) Single-copy insertion of transgenes in Caenorhabditis elegans. Nat Genet 40: 1375–1383. doi:10.1038/ng.248.

2. Hobert O (2002) PCR fusion-based approach to create reporter gene constructs for expression analysis in transgenic C. elegans. Biotechniques 32: 728–730.
